# Effects of supplementation with vitamin D_3_ on growth performance, lipid metabolism and cecal microbiota in broiler chickens

**DOI:** 10.3389/fvets.2025.1542637

**Published:** 2025-02-06

**Authors:** Jiawei Li, Ximei Li, Jiamin Tian, Linna Xu, Yan Chen, Susu Jiang, Guohua Zhang, Jianxiong Lu

**Affiliations:** ^1^School of Life Sciences and Engineering, Northwest Minzu University, Lanzhou, China; ^2^Gansu Provincial Animal Husbandry Technology Popularization Station, Lanzhou, China

**Keywords:** broiler chicken, vitamin D_3_, growth performance, lipid metabolism, cecal microbiota

## Abstract

Lower intramuscular fat (IMF) and excessive abdominal fat reduce carcass quality in broilers. The study aimed to investigate the effects of dietary VD_3_ on growth performance, lipid metabolism and cecal microbiota in broilers over an 84-d feeding experiment. One-day-old male *Luhua* broilers (210) were randomly assigned to control (basal diet) and VD group (basal diet supplemented with 3,750 IU/kg VD_3_). Samples were collected after a 12-h fasted feeding on days 28, 56, and 84. Supplementary VD_3_ significantly enhanced average daily gain (ADG) in broilers aged 57-84 d and 1-84 d, and increased leg muscle rate and fat content in breast and leg muscles and reduced abdominal fat rate of broilers at 84 d. VD_3_ increased TG and glycogen content in the liver of 28- and 84-d-old broilers, serum TG and VLDL-C content at 56 and 84 d, and TC, HDL-C and LDL-C at 84 d. VD_3_ increased mRNA expressions of genes related to *de novo* lipogenesis (DNL) (*mTOR*, *SREBP-1c*, *FAS* and *ACC*), lipid oxidation (*AMPK*, *PPARα*, *CPT-1α* and *ACO*) and lipid transport (*ApoB* and *MTTP*), and FAS, ACC and CPT1 enzyme activities in the liver. However, mRNA levels of genes involved in DNL and cellular lipid uptake (*LPL* and *FATP1*) and LPL activity were decreased in abdominal adipose tissue, and that of genes involved in lipid oxidation and lipolysis (*HSL* and *ATGL*) was increased by VD_3_. *LPL* and *FATP1* expression in breast and leg muscles was increased by VD_3_. Moreover, VD_3_ increased the abundance of cecum *Bacteroides* at 28 and 84 d, *Rikenellaceae_RC9_gut_group* and *Faecalibacterium* at 56 and 84 d, and *Lachnoclostridium* at 84 d. These bacteria were correlated with increased DNL, lipid oxidation and lipid transport in liver, and cellular lipid uptake in muscle, as well as decreased DNL and cellular lipid uptake, and increased lipid oxidation and lipolysis in abdominal adipose tissue. Altogether, supplementary VD_3_ in basal diet improved growth performance, increased IMF, and reduced abdominal fat rate, which is significant for enhancing feed utilization and improving the carcass quality of broilers. The regulation of VD_3_ on lipid metabolism could was associated with variation in cecal microbiota composition.

## 1 Introduction

Chicken meat is the second most-consumed meat worldwide by humans. With continuous advancements in breeding and high-density feeding, the chicken production has significantly increased. However, a relatively lower intramuscular fat (IMF) content negatively impacts the meat quality (1), while excessive accumulation of abdominal fat reduces the carcass quality and feed conversion rate in broiler chickens (2), presenting a challenging issue in modern broiler production systems. Many factors, such as genetics, rearing systems, and dietary energy balance, influence fat deposition in broilers. The regulation of micronutrients on lipid metabolism is recognized as a crucial pathway for controlling body fat deposition and energy distribution in animals.

Vitamin D (VD) is a class of steroid derivatives that exist in multiple forms due to variations in their side chain structures. VD exhibits the functional characteristics of both vitamins and hormones, with ergocalciferol (vitamin D_2_, VD_2_) and cholecalciferol (vitamin D_3_, VD_3_) serving as the primary functional substances in animals. VD_3_ is the primary form of VD used by chickens, as they cannot efficiently metabolize plant-derived VD_2_ (3). VD_3_ can be synthesized endogenously by the skin at sufficient UV-B exposure, but its main source is dietary intake (4, 5). It is well known that VD plays a key regulatory role in the absorption, utilization and metabolic homeostasis of calcium and phosphorus, as well as in bone development and health of animals (3, 6, 7). However, recent studies have shown that adipose tissue, as a main storage site for VD, also expresses VD hydroxylase, catalyzing the generation of the active form of VD, 1,25-dihydroxyvitamin D_3_ [1,25 (OH)_2_D_3_] (8). This active form could influence biological functions, such as adipogenesis, in non-classical target tissues through autocrine, paracrine, or endocrine pathways. VD regulated various biological processes in its active form in adipose tissue through the mediation of the vitamin D receptor (VDR), including the proliferation and differentiation of adipocyte, lipogenesis, and the expression of adipokines and inflammatory responses (9, 10).

VD_3_ has been reported to improve glucose homeostasis and maintain metabolic balance in humans and rats (11, 12). VD_3_ enhanced glucose uptake in 3 T3-L1 cells by promoting AMPK phosphorylation and the expression and translocation of glucose transporter 4 (GLUT4), which was supported by *in vivo* experiments showing that VD_3_ increased glucose uptake in both adipocytes and adipose tissue of mice (13). VD_3_ reduced triglyceride (TG) levels in liver by inhibiting lipogenesis through the downregulation of sterol regulatory element binding protein 1-c (SREBP1-c) and fatty acid synthase (FAS) expression, while simultaneously enhancing β-oxidation of fatty acids by increasing the expression of carnitine palmitoyl transferase 1α (CPT-1α) and peroxisome proliferator-activated receptor (PPAR) α in mice (14). Supplementation of VD_3_ alleviated liver steatosis in high-fat-induced obese mice (12) and mitigated hepatic steatosis by regulating fatty acid uptake and β-oxidation through PPARα signaling pathway in mice (15). 1,25(OH)_2_D_3_ reduced lipid storage and lipid droplet aggregation by decreasing the expression of *FAS* and *PPARγ*, while increasing the expression of *CPT-1α*, *PPARα* and hormone-sensitive lipase (*HSL*) in 3 T3-L1 adipocytes (16). On the contrary, VD_3_ deficiency significantly decreased the expression of β-oxidation-related genes, such as *CPT1α*, PPARγ coactivator-1 α (*PGC1α*), and *PPARα*, as well as protein level of AMP-activated protein kinase (AMPK) in obese rats (17). Additionally, VD_3_ influenced gut microbiota to modulate the host’s metabolism through VDR, which was abundantly expressed in the gut (18). Dietary supplementation with VD_3_ reduced serum levels of HDL-C, TG, and TC, increased the abundance of gut microbiota, and improved carbohydrate and amino acid metabolism in mice (19). Zhang et al. (20) showed that VD_3_ promoted the restoration of gut microbiota dysbiosis and inhibited lipid accumulation caused by a high-fat diet by increasing the abundance of *Lactobacillus* while decreasing that of *Oscillibacter* and *Flavonifractor* in the gut of rats.

Previous studies have shown that VD_3_ regulated glucose and lipid metabolism in humans and rodents, and this effect was enduring and persistent (21, 22). It has also been demonstrated that VD_3_ was beneficial for bone quality (7), immunity (9), and antioxidants (23), which could be related to the gut microbiota. While VD_3_ supplementation improved growth and carcass performance, affected meat color, and decreased abdominal fat percentage (24, 25), current studies have not clarified the effect of VD_3_ on lipid metabolism and its relationship with the gut microbiota in broilers. In this study, we investigated the effects of VD_3_ supplementation on a basal diet on the growth performance and carcass quality in *Luhua* broilers, a medium-growing strain that is popular locally because of the chewy texture of the meat. Due to its lengthy growth period and significant fat deposition capability, this broiler is an excellent subject for studying fat deposition. Furthermore, we systematically explored the mechanism by which supplementary VD_3_ affected fat accumulation by investigating variations in lipid metabolism in the liver and adipose tissue, lipid transport, and cecal microbiota in the broilers.

## 2 Materials and methods

### 2.1 Ethics statement

All procedures involving in animals were performed following the Regulations for the Administration of Affairs Concerning Experimental Animals (Ministry of Science and Technology, China, 2004) and were approved and supervised by the Northwest Minzu University Animal Care and Use Committee (Permit No. xbmu-sm-20210130).

### 2.2 Experimental design and animal management

A total of 210 healthy one-day-old male *Luhua* chicken broilers with similar body weight were randomly divided into two groups, each with 7 replicates and 15 chickens per replicate. The broilers in the control group (CON group) were fed a basal diet, while the broilers in the VD_3_ group (VD group) received the basal diet supplemented with 3,750 IU/kg of VD_3_ (24). The VD_3_ product was supplied by Guangzhou Applon Biotechnology Co., Ltd. (Guangzhou, China). The basal diet was formulated according to the broiler Feeding Standard in China (GB/T 5916-2020) and was detailed in Table 1.

**Table 1:** Formulation and proximate composition of the basal diets (as-fed basis, %).

Items	1–28 days of age	29–56 days of age	57–84 days of age
Ingredients			
Corn	51.8	50.7	58.7
Soybean oil	2.5	4.6	5.2
Soybean meal	28	26	23
Cottonseed meal	8	7.6	7
Rapeseed meal	0	8	3.36
Corn gluten meal	6	0	0
CaHPO_4_	1.5	0.82	0.6
NaCl	0.35	0.34	0.3
*L*-Lys HCL	0.2	0.36	0.2
*DL*-Met	0.1	0.1	0.1
Cys	0.08	0.08	0.03
Premix^1^	1.47	1.4	1.51
Total	100	100	100
Nutrient levels^2^			
ME/(MJ/kg)	18.53	16.91	14.65
CP	21.00	18.00	17.00
Ca	1.04	1.10	0.86
TP	0.89	0.85	0.62
Lys	1.024	1.054	0.900
Met	0.435	0.349	0.292

1 The premix provided the following per kg of diets: 1–28 days of age, VA 12,000 IU, VD_3_ 3,500 IU, VE 60 IU, VK_3_ 4 mg, VB, 2.5 mg, VB_1_ 10 mg, VB6 6 mg,VB_6_ 6 mg, VB_12_ 8 μg, D-pantothenic acid 40 mg, nicotinic acid 75 mg, folic acid 10 mg, biotin 0.8 mg, choline 700 mg, Zn 90 mg, Fe 110 mg, Cu 20 mg, Mn 100 mg, I 0.5 mg, Se 0.3 mg; 29–56 days of age, VA 11,000 IU, VD_3_ 3,300 IU, VE 55 IU, VK_3_ 3.5 mg, VB_1_ 6 mg, VB_2_ 10 mg, VB_6_ 5 mg, VB_12_ 6 μg, D-pantothenic acid 30 mg, nicotinic acid 70 mg, folic acid 9 mg, biotin 0.7 mg, choline 600 mg, Zn 80 mg, Fe 100 mg, Cu 17 mg, Mn 90 mg, I 0.5 mg, Se 0.3 mg; 57–84 days of age, VA 10,000 IU, VD_3_ 3,000 IU, VE 50 IU, VK_3_ 3.0 mg, VB1 2 mg, VB_2_ 14 mg, VB_6_ 5 mg, VB_12_ 4 μg, D-pantothenic acid 20 mg, nicotinic acid 60 mg, folic acid 7 mg, biotin 0.6 mg, choline 600 mg, Zn 70 mg, Fe 100 mg, Cu 15 mg, Mn 85 mg, I 0.5 mg, Se 0.3 mg.

2 Nutrient levels were all calculated values.

The trial was conducted at ShunHe Broiler Breeding Farm (Lanzhou, China). The broilers were raised in flat net-rearing system. Each replication was reared separately in a single pen (70 cm from the concrete floor) consisting of a stainless-steel frame with a flat wire net covered. Each pen was 200 × 100 cm with a round feeder pan and nipple drinkers (5 nipple drinkers in middle of pen). All broilers were housed in a windowed house with a controlled ventilation regime and a wet curtain cooling system. The temperature inside the house was maintained at 35 ~ 37°C with a relative humidity (RH) of 50% during the first week, gradually decreasing by 2°C each week until the coop temperature reached 24°C with RH at 45%. The lighting regime was 23-h light/1-h dark daily for chicks aged 1–28 days, and 16-h light/8-h dark daily for broilers aged 29–84 days. The experiment lasted 84 days. The broilers had ad libitum access to feed and water. The broilers received the Newcastle disease vaccine and the infectious bursal polyvalent vaccine on day 7 and 14 of the experiment, respectively.

### 2.3 Growth performance and carcass traits

The body weight (BW) of broilers was measured every 14 days after fasting for 12 h, and the feed intake on a pen basis was recorded daily. The average daily gain (ADG), average daily feed intake (ADFI) and feed conversion rate (FCR, feed/gain) were calculated. Mortality was recorded as it occurred.

On the last day of the experiment (day 84), two broilers with a BW close to the average in each replicate were selected and killed after fasting for 12 h. Following bleeding and plucking, the carcass was individually weighed. Subsequently, the birds were eviscerated, and weights were measured. The half-eviscerated carcass weight was calculated by excluding the trachea, esophagus, intestines, spleen, pancreas, gallbladder, reproductive organs, and gizzard contents and corneum from the carcass. The eviscerated weight was calculated by removing the heart, liver, proventriculus, gizzard, lungs, and abdominal fat from the half-eviscerated carcass. The rates of dressing, half-eviscerated carcass, eviscerated carcass, breast muscle, thigh muscle (thigh and drumstick), abdominal fat (fat around the abdomen), and liver were calculated as relative weight to the live BW.

### 2.4 Sample collection

On the morning of the last day of the starter stage (days 1 to 28), the grower stage (days 29 to 56), and the finisher stage (days 57 to 84), that was on days 28, 56, and 84, after a 12-h fasted feeding, two broilers in each pen (i.e., 14 birds per group) were randomly selected for sampling. 5 mL of blood samples were collected from the wing vein of each individual, and serum was collected through centrifuging at 3,000 rpm for 10 min. Afterwards, the broilers were slaughtered and quickly dissected, and approximately 5 g samples of liver, abdominal adipose tissue, and leg and breast muscle were collected. The cecal contents were carefully collected, homogenized using a sterile spatula, and transferred to CryoPure Tubes (Sarstedt AG + Co., Nümbrecht, Germany). The samples collected above were snap-frozen in liquid nitrogen and stored at −80°C until analysis.

### 2.5 Determination of fat content

Approximately 2.0 g of tissue samples were freeze-dried using a vacuum freeze dryer and then ground to a homogeneous powder. The fat (ether extract, EE) in the tissue samples was extracted using a Soxhlet extractor (SOX406, Hanon Advanced Technology Group Co., Ltd., Shandong, China) (GB 5009.6–2016, China). Fat content was expressed as a percentage of fat in fresh tissue samples.

### 2.6 Biochemical parameter analysis

Nine times the cooled physiological saline was added to 1 g of liver and adipose tissue samples, which were ground in a glass homogenizer to prepare a tissue homogenate. The homogenate was centrifuged at 4°C at 3000 × *g* for 10 min, and the supernatant was collected. The supernatant and serum samples were analyzed for triglyceride (TG), total cholesterol (TC), high-density lipoprotein cholesterol (HDL-C), low-density lipoprotein cholesterol (LDL-C), very low-density lipoprotein cholesterol (VLDL-C), free fatty acid (FFA), glycogen and glucose content using an automatic biochemical analyzer (BS-200; Shenzhen Mindray Bio-Medical Electronics Co., Ltd., Shenzhen, China). The contents of insulin and VD, and the activities of FAS, acetyl-CoA carboxylase (ACC), CPT-1α and lipoprotein lipase (LPL), were determined using chicken-specific enzyme-linked immunosorbent assay (ELISA) kits following the manufacturer’s protocol (Solarbio Science & Technology Co., Beijing, China).

### 2.7 Real-time quantitative polymerase chain reaction

Total RNA were extracted using TRIzol reagent and cDNA was synthesized with a reverse transcription kit according to the manufacturer’s protocol (TaKaRa Biotechnology, Dalian, China). Real-time RT-PCR was performed on a fluorescent quantitative detection system (FQD-96A; Hangzhou Bioer Technology Co., China). Amplification was conducted in a total volume of 10 μL containing 5 μL of SYBR Green PCR Master Mix (TaKaRa Biotechnology, Dalian, China), 0.4 μL of each forward and reverse primer, 1 μL of cDNA, and 3.2 μL of ddH_2_O. PCR reaction procedure: pre-denaturation at 95°C for 30 s; followed by 40 cycles of 95°C for 5 s, 60°C for 30 s, and 72°C for 30 s. The relative expression of each target gene was expressed using comparative threshold cycle (2^−ΔΔCt^) method and β-*actin* as an internal control. The specificity of the PCR amplification was verified with melting curve analysis. The information of primers used in this study was listed in Table 2.

**Table 2:** Primer sequences for RT-PCR.

Gene	Primer sequences, 5′–3′	Gene Bank No.
*β-actin*	F: TCCACCGCAAATGCTTCTAA	NM_205518.2
	R: AAGCCATGCCAATCTCGTCT	
*Mammalian target of rapamycin*, *mTOR*	F: CCCGCTGTCCAAGGTTTCT	XM-040689168.2
	R: CTATTTGGATTGCCTTGACCC	
*Sterol regulatory element binding protein 1-c SREBP-1c*	F: GTCCCGAGGGAGACCATCTA	AY029224
	R: CAACGCATCCGAAAAGCA	
*Fatty acid synthase*, *FAS*	F: AGCTGAAGGCTGCTGACAAG	NM_205155
	R: CCTCCAATAAGGTGCGGTSA	
*Acetyl-CoA Carboxylase*, *ACC*	F: TCCTGATTCCCATTTACCACC	NM_205505
	R: TTTCCAGTCCAGAATGTCCGT	
*AMP-activated protein kinase*, *AMPK*	F: CGAAGTGGCATTTGGGGATA	NM_001039603
	R: CCGTCGAACACGCAAGTAGTA	
*Peroxisome proliferator-activated receptor α*, *PPARα*	F: TTTTGTCGCTGCCATCATTT	NM-001001464.1
	R: GGAGAAGTTTCGGGAAGAGGA	
*Carnitine palmitoyltransferase 1α*, *CPT-1α*	F: TAGAGGGCGTGGACCAATAA	NM_001012898.1
	R: GAGCAGGATGGCATGGATAA	
*Acyl-CoA oxidase*, *ACO*	F: AATGCTGGTATTGAGGAATGTCG	NM_001006205.1
	R: TGCAGGATGGGGTGAACGT	
*Apolipoprotein B*, *ApoB*	F: GCTTAGAATAGATGTGCCGTTTG	NM-001044633.2
	R: CCCATTTCCTGGTGCCTTGT	
*Microsomal triglyceride transfer protein*, *MTTP*	F: AGTTTTCACAGTACCCCTTCCTAG	NM-001109784.3
	R: TCCAACATTTCTGCTTTCCCT	
*Sterol regulatory element binding protein 2*, *SREBP2*	F: CGAAGTCCCTGGAGATGTCTG	XM-015289037.4
	R: GCACCGCTGCTCATGTTGA	
*Cholesterol 7α-hydroxylase*, *CYP7A1*	F: TCTGTTGCCAGGTGATGTTTG	NM-001001753.2
	R: TGGGCACTCTTGAATAGATGGATAG	
*Lipoprotein lipase*, *LPL*	F: GAAGGGTTTGAAGGTAGGCATT	NM-205282.2
	R: ACCACCTCCACATTTTGTCTTG	
*Fatty acid transport protein1*, *FATP1*	F: CAGCAATCGCAGATCCTAAAA	NM-001398142.1
	R: CAACCTGGGGTGAAAGACG	
*Hormone-sensitive lipase*, *HSL*	F: GATTTCTCAGCCTTTCCCCTCT	XM-040657096.1
	R: CCATCCCCATAGCACCCAAT	
*Adipose triglyceride lipase*, *ATGL*	F: GCTCAGGTAAAGAAAGTGCAGGTC	NM-001113291.2
	R: GCAAGAACGTCAAGGAAATTGTG	

### 2.8 Cecal microbial diversity analysis

Bacterial genomic DNA was isolated from the cecal contents using the TGuide S96 kit (DP812; Tiangen Biotech Co., Beijing, China) following the manufacturer’s instructions. The purity and quality of the DNA were verified in 0.8% agarose gels. Subsequently, the full-length 16S rRNA gene was amplified using the primers (27F: AGRGTTTGATYNTGGCTCAG and 1492 R: TASGGHTACCTTGTTASGACTT). The sequencing libraries (SMRT Bell) were constructed using purified PCR products and sequenced on PacBio Sequel II platform (Biomarker-Technologies Co., China). SMRT-Link v8.0 was used to correct the original subreads to obtain Circular Consensus Sequencing (CCS) sequence. The CCS sequences were identified using Lima v1.7.0 software and filtered with cutadapt 1.9.1 software, and the non-chimeric CCS were obtained by the DADA2 method of QIIME2 2020.6 software. BMK Cloud^1^ was used for Alpha diversity, Beta diversity, and microbial composition analysis.

### 2.9 Statistical analysis

Statistical analysis was performed using a two-tailed Student’s *t*-test in the SPSS software (version 26.0; IBM Corp., Armonk, NY, USA), with the results expressed as± Standard Error of Means (SEM). Statistical significance was established at *p* < 0.05. Spearman correlation analysis was performed using R version 3.5.1 to evaluate the correlations between cecal biomarker bacterial genera and significantly different lipid metabolism indicators. GraphPad Prism 8.02 (GraphPad, Inc. La Jolla, CA, USA) was used for data mapping charts.

## 3 Results

### 3.1 Growth performance, carcass traits and fat deposition

The broilers remained in good health throughout the experiment. The effect of the supplementary VD_3_ on growth performance was presented in Table 3. There was no significant difference (*p* > 0.05) in the BW of broilers at 1, 28, and 56 days of age among the groups; however, the BW of broilers at 84 days of age significantly increased in the VD group compared to the CON group (*p* < 0.05). VD_3_ supplementation did not significantly affect (*p* > 0.05) the ADFI and FCR (feed/gain) of broilers at any growth stages, nor the ADG of broilers aged 1–28 days and 29–56 days. However, supplementary VD_3_ significantly increased (*p* < 0.05) the ADG in broilers aged 57–84 days and 1–84 days.

**Table 3:** Effects of VD_3_ supplementation on growth performance of broilers.

	CON Group	VD Group	SEM	*p*-value
Day 1–28				
BW (1 d), g	34.87	34.98	0.11	0.630
BW (28 d), g	704.2	712.86	3.83	0.276
ADFI, g/d	44.53	43.68	0.41	0.308
ADG, g/d	23.90	24.21	0.14	0.279
FCR (feed/gain)	1.86	1.80	0.02	0.142
Day 29–56				
BW (56 d), g	2076.28	2126.83	14.33	0.108
ADFI (g/d)	133.61	128.24	2.12	0.234
ADG (g/d)	49.01	50.50	0.46	0.140
FCR (feed/gain)	2.73	2.54	0.05	0.072
Day 57–84				
BW (84 d), g	3472.07	3585.83^*^	21.24	0.023
ADFI (g/d)	167.70	171.27	2.85	0.545
ADG (g/d)	49.85	52.11^*^	0.45	0.030
FCR (feed/gain)	3.37	3.29	0.17	0.574
Day 1–84				
ADFI (g/d)	115.28	114.39	1.43	0.767
ADG (g/d)	40.92	42.27^*^	0.25	0.024
FCR (feed/gain)	2.82	2.71	0.04	0.168

Values with superscripts “^*^” indicate significant difference (*p* < 0.05).

The carcass performance of broilers at 84 days of age and the fat deposition at 28, 56 and 84 days of age were evaluated (Table 4). Broilers fed a diet supplemented with VD_3_ exhibited a significantly higher (*p* < 0.05) leg muscle rate and liver organ index compared to the CON group at 84 days of age. However, there were no significant differences (*p* > 0.05) in the other tested traits.

**Table 4:** Effects of VD_3_ supplementation on the carcass performance and fat deposition in broilers (%).

Items	CON Group	VD Group	SEM	*p*-value
Day 28				
Liver organ index	26.48	28.66	0.51	0.054
Abdominal fat rate	2.41	2.26	0.06	0.406
Fat content in liver	4.80	4.82	0.13	0.245
Fat content in breast muscle	1.70	1.88	0.06	0.211
Fat content in leg muscle	3.71	4.08	0.12	0.188
Day 56				
Liver organ index	17.55	19.41	0.44	0.067
Abdominal fat rate	2.01	1.93	0.05	0.295
Fat content in liver	5.55	5.83	0.19	0.175
Fat content in breast muscle	2.52	2.75	0.08	0.174
Fat content in leg muscle	5.77	6.17	0.11	0.122
Day 84				
Dressing rate	92.78	93.73	0.39	0.285
Half-eviscerated rate	84.55	86.06	0.56	0.203
Eviscerated rate	66.19	66.39	0.61	0.870
Breast muscle rate	15.39	15.41	0.21	0.950
Leg muscle rate	23.57	26.98^*^	0.49	0.004
Liver organ index	15.36	18.76^*^	0.63	0.020
Abdominal fat rate	3.05	2.57^*^	0.08	0.010
Fat content in liver	8.71	9.74^*^	0.18	0.019
Fat content in breast muscle	4.07	5.29^*^	0.15	0.017
Fat content in leg muscle	10.08	11.13^*^	0.14	0.022

Values with superscripts “^*^” indicate significant difference (*p* < 0.05).

Compared to the CON group, the supplementary VD_3_ significantly (*p* < 0.05) reduced the abdominal fat rate and increased the fat content in the liver, breast muscle, and leg muscle of broilers at 84 days of age, with no significant effects observed at 28 and 56 days of age (*p* > 0.05). This indicated that supplementation with VD_3_ in diet had varying effects on fat deposition in different tissues of broilers.

### 3.2 Parameters related to lipid metabolism in liver and serum

**Figure 1:**
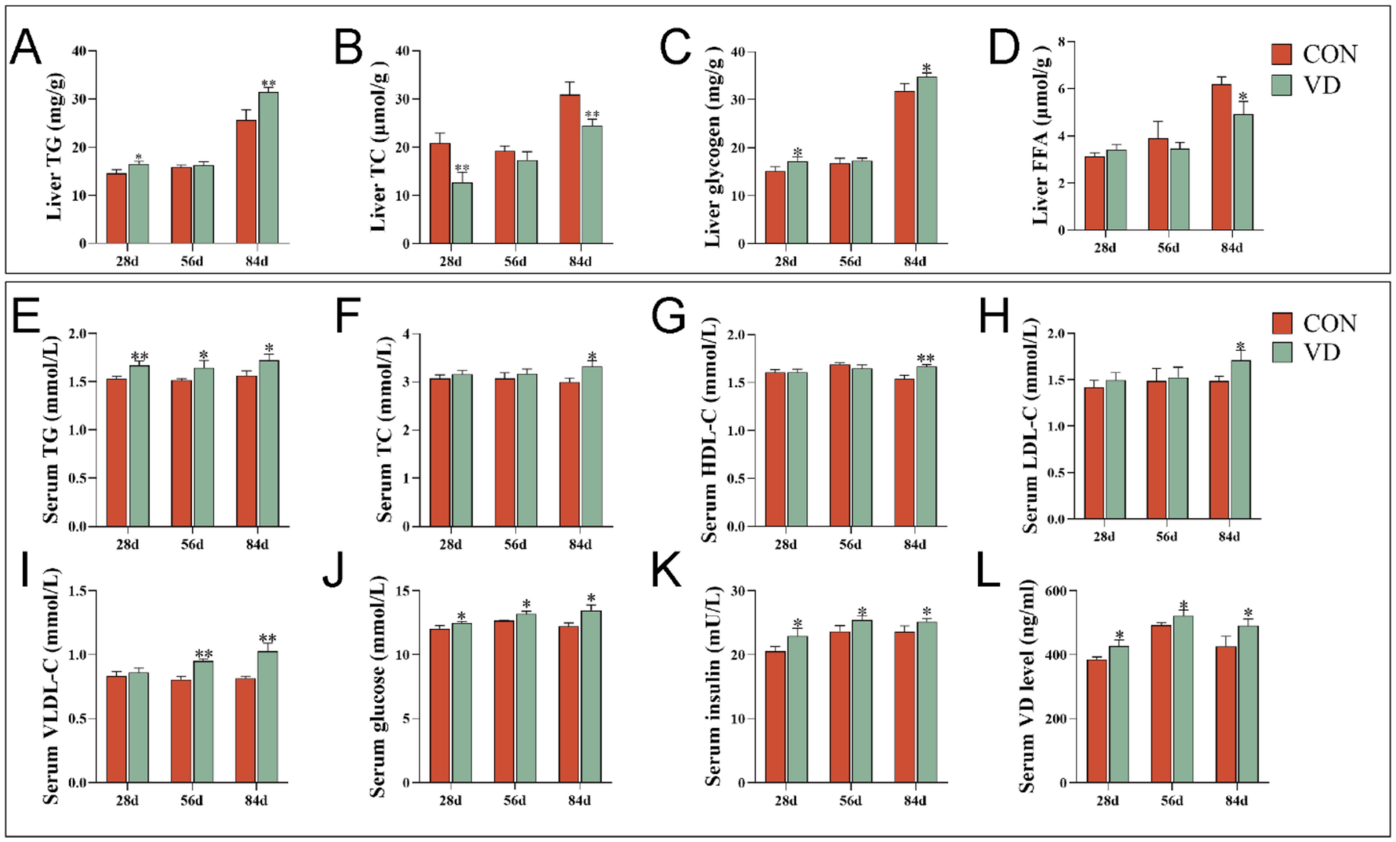
Effect of dietary VD_3_ supplementation on the liver and serum parameters in broilers. **(A)** The content of TG in the liver; **(B)** TC content in the liver; **(C)** liver glycogen content in the liver; **(D)** FFA content in the liver; **(E)** TG content in serum; **(F)** TC content in serum; **(G)** HDL-C content in serum; **(H)** LDL-C content in serum; **(I)** VLDL-C content in serum; **(J)** glucose content in serum; **(K)** insulin content in serum; **(L)** VD content in serum. Data are presented as mean ± SEM, *n* = 14. *p < 0.05; **p < 0.01.

The supplementary VD_3_ significantly increased (*p* < 0.05) the content of TG and glycogen while decreasing (*p* < 0.05) the TC content in the liver of 28- and 84-day-old broilers, and also reduced (*p* < 0.05) the FFA content in the liver of 84-day-old broilers (Figures 1A–D). However, supplementary VD_3_ did not affect (*p* > 0.05) these parameters in the liver of 56-day-old broilers. Notably, all these parameters were significantly higher (*p* < 0.05) in 84-day-old broilers compared to those that were 28 and 56 days old.

The supplementary VD_3_ significantly increased (*p* < 0.05) the content of TG, glucose, insulin and VD in serum of 28-, 56- and 84-day-old broilers, as well as the content of TC, HDL-C and LDL-C in serum of 84-day-old broilers, and the VLDL-C content in serum of 56- and 84-day-old broilers (Figures 1E–L). This indicates that at different growth stages, VD_3_ increased serum lipid levels and promoted lipid transport in the blood, especially in the middle and late stages of growth.

### 3.3 The expressions of genes and the activities of enzymes related lipid metabolism in the liver

**Figure 2:**
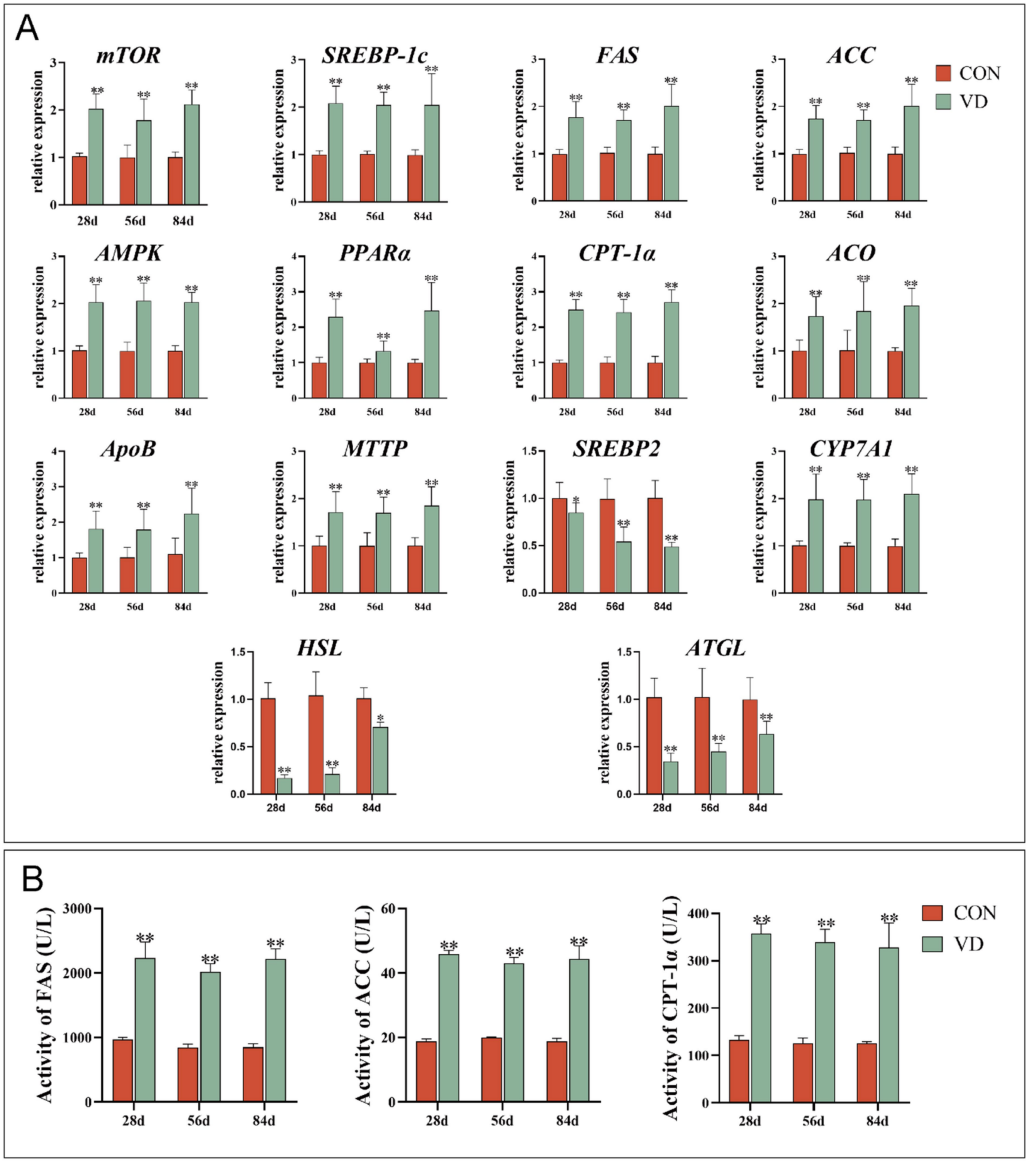
Effect of dietary VD_3_ supplementation on the expression of genes and the activity of enzymes involved in lipid metabolism in the liver. **(A)** The expression of lipid metabolism genes. **(B)** The activity of FAS, ACC and CPT-1α. Data are presented as mean ± SEM (*n* = 14). *p < 0.05; **p < 0.01.

Considering that the liver is the primary site for lipid biosynthesis in poultry, the expression of genes and the activity of enzymes involved in lipid metabolism in the liver were detected (Figure 2). For broilers at 28, 56 and 84 d of age, supplementary VD_3_ increased significantly (*p* < 0.05) the mRNA expression of the lipogenic gene *mTOR*, *SREBP-1c*, *FAS* and *ACC*, the lipid oxidizing gene *AMPK*, *PPARα*, *CPT-1α* and *ACO*, the VLDL assembly protein encoding-gene *ApoB* and *MTTP*, the cholesterol catabolism gene *CYP7A1*. Conversely, the expression of the cholesterol synthesis gene *SREBP2*, and the lipolytic gene *HSL* and *ATGL* was significantly decreased (*p* < 0.01) by the supplementary VD_3_ (Figure 2A). Furthermore, the supplementary VD_3_ significantly increased (*p* < 0.01) the activity of FAS, ACC and CPT-1α in the liver of broilers at 28, 56 and 84 days of age (Figure 2B).

### 3.4 The expressions of genes and the activity of enzyme related lipid metabolism in extrahepatic tissues

**Figure 3:**
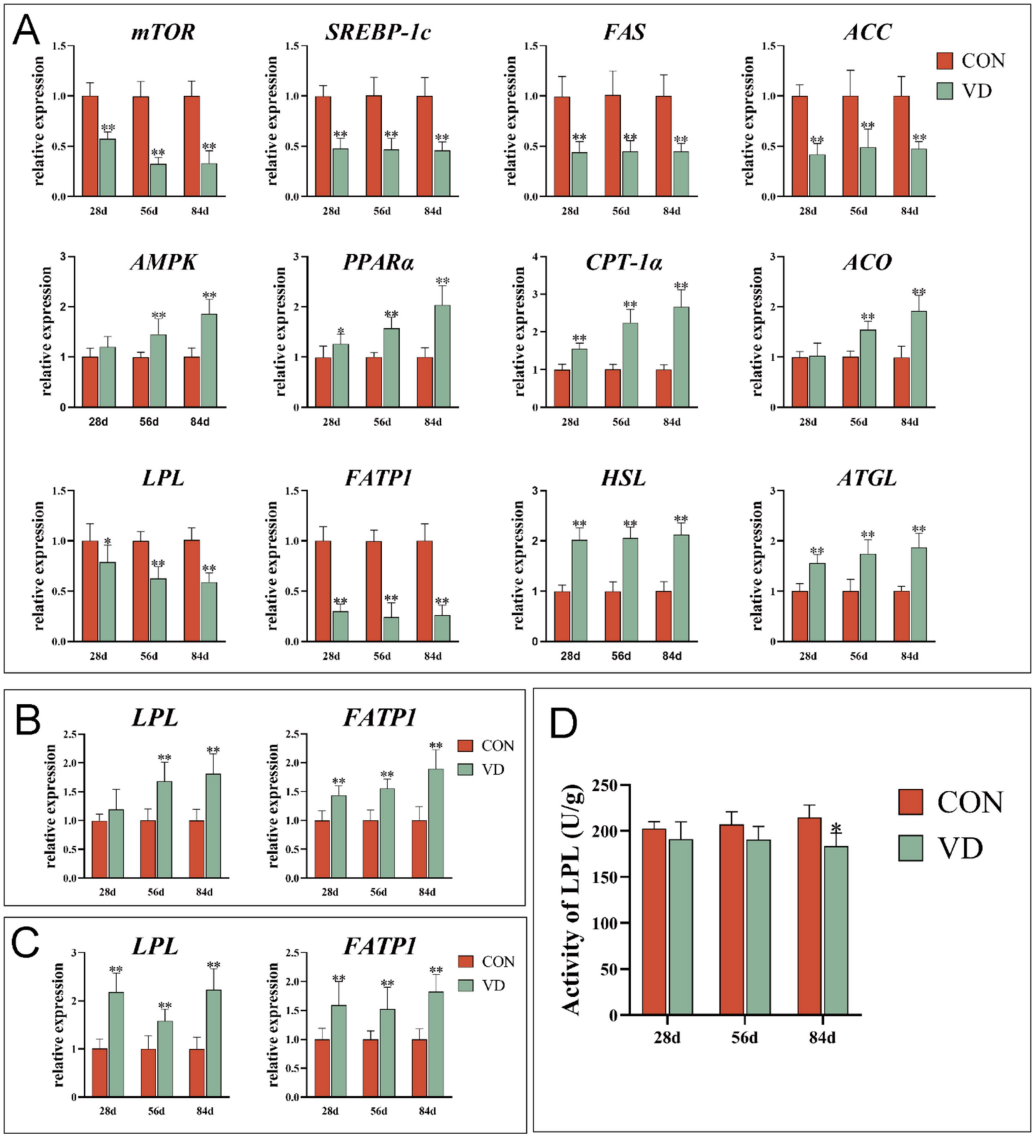
Effect of dietary VD_3_ supplementation on gene expression and enzyme activity related to lipid metabolism in extrahepatic tissues. **(A)** The expression of genes in abdominal adipose tissue. **(B)** The expression of genes in breast muscle. **(C)** The expression of genes in leg muscle. **(D)** The LPL activity in abdominal adipose tissue. Data are presented as mean ± SEM (*n* = 14). *p < 0.05; **p < 0.01.

To understand the effects of supplementary VD_3_ on lipid metabolism in extrahepatic tissues, the mRNA expression of lipid metabolism genes in abdominal adipose tissue, breast and leg muscle tissues was detected (Figure 3). Supplementary VD_3_ decreased significantly (*p* < 0.01) the expression of lipogenic gene *mTOR*, *SREBP-1c*, *FAS* and *ACC*, lipid uptake gene *LPL* and *FATP1*, and increased (*p* < 0.05) the expression of the lipolytic gene *HSL* and *ATGL*, and lipid oxidizing gene *PPARα* and *CPT-1α* in abdominal adipose tissue of broilers at 28, 56 and 84 days of age, as well as *AMPK* and *ACO* in broilers at 56 and 84 days of age (Figure 3A). Additionally, the LPL activity in the abdominal adipose tissue of broilers at 84 days of age was significantly decreased (*p* < 0.05) by supplementary VD_3_ (Figure 3D).

The supplementary VD_3_ significantly increased (*p* < 0.01) the *LPL* expression in breast muscle of broilers at 56 and 84 days of age, and the expression of *LPL* in leg muscle and *FATP1* in both breast muscle and leg muscle of broilers at 28, 56 and 84 days of age (Figures 3B,C).

### 3.5 Variation in cecal microbiota diversity

#### 3.5.1 Alpha diversity and beta diversity

**Figure 4:**
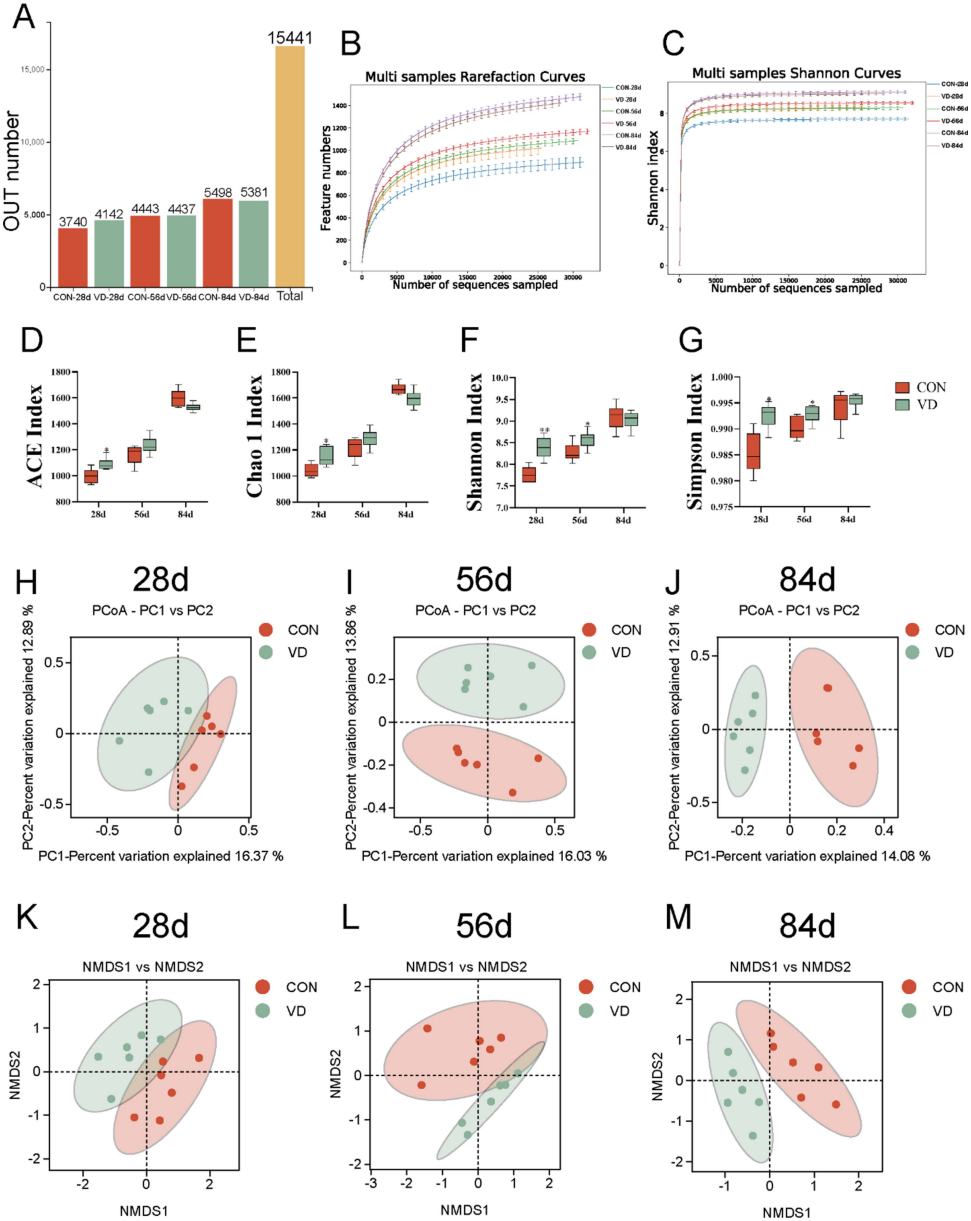
Effect of dietary VD_3_ supplementation on cecal microbiota diversity in broiler. **(A)** OUT number. **(B)** Rarefaction curve. **(C)** Shannon-Wiener curve. **(D)** ACE index. **(E)** Chao1 index. **(F)** Shannon index. **(G)** Simpson index. **(H–J)** PCoA based on OTUs at 28, 56 and 84 days of age, respectively. The percent variation explained by each principal coordinate is indicated on the axes. (**K–M**) NMDS based on OTUs at 28, 56 and 84 days of age. *p < 0.05; **p < 0.01.

A total of 15,411 operational taxonomic units (OTUs) were identified in 36 cecal content samples of broilers from the CON and VD groups with 97% confidence interval (Figure 4A). The Rarefaction curves and Shannon curves of cecal samples flattened out with the increasing numbers of sequences sampled, indicating that the sample size was reasonable, and the sequencing depth was sufficient for all samples based on a saturated trend (Figures 4B,C).

Alpha diversity analysis was used to assess the richness and diversity of gut microbiota (26). Both the ACE and Chao1 indexes represent the richness of the microbial community. Dietary VD_3_ supplementation significantly increased ACE and Chao1 indexes in 28-day-old broilers compared to the CON group, while showing no effects in 56- and 84-day-old broilers (Figures 4D,E). Both Shannon and Simpson indexes were significantly higher in the VD group than in the CON group at 28 and 56 days of age (*p* < 0.05), while there was no significant difference at 84 days of age (Figures 4F,G). These results indicated that VD_3_ influenced α-diversity of the cecum microbiota in broiler chickens during the early growth stage.

Beta diversity, a measure of variance in taxa composition between sampling sites (27), was visualized by plotting the distances between samples using principal coordinates analysis (PCoA) and non-metric multi-dimensional scaling (NMDS) biplot. The PCoA and the NMSD analysis exhibited that at 28 days of age, there was not a complete separation in the cecal microbial community between the CON and VD groups (Figures 4H,K). At 56 and 84 days of age, a distinct separation between the groups was observed, with samples clustering within each group (Figures 4I,J,L,M). These results indicated that supplementary VD_3_ had an impact on the composition of cecal microbiota in broilers.

#### 3.5.2 Variation in cecal microbiota composition at the phylum level

**Figure 5:**
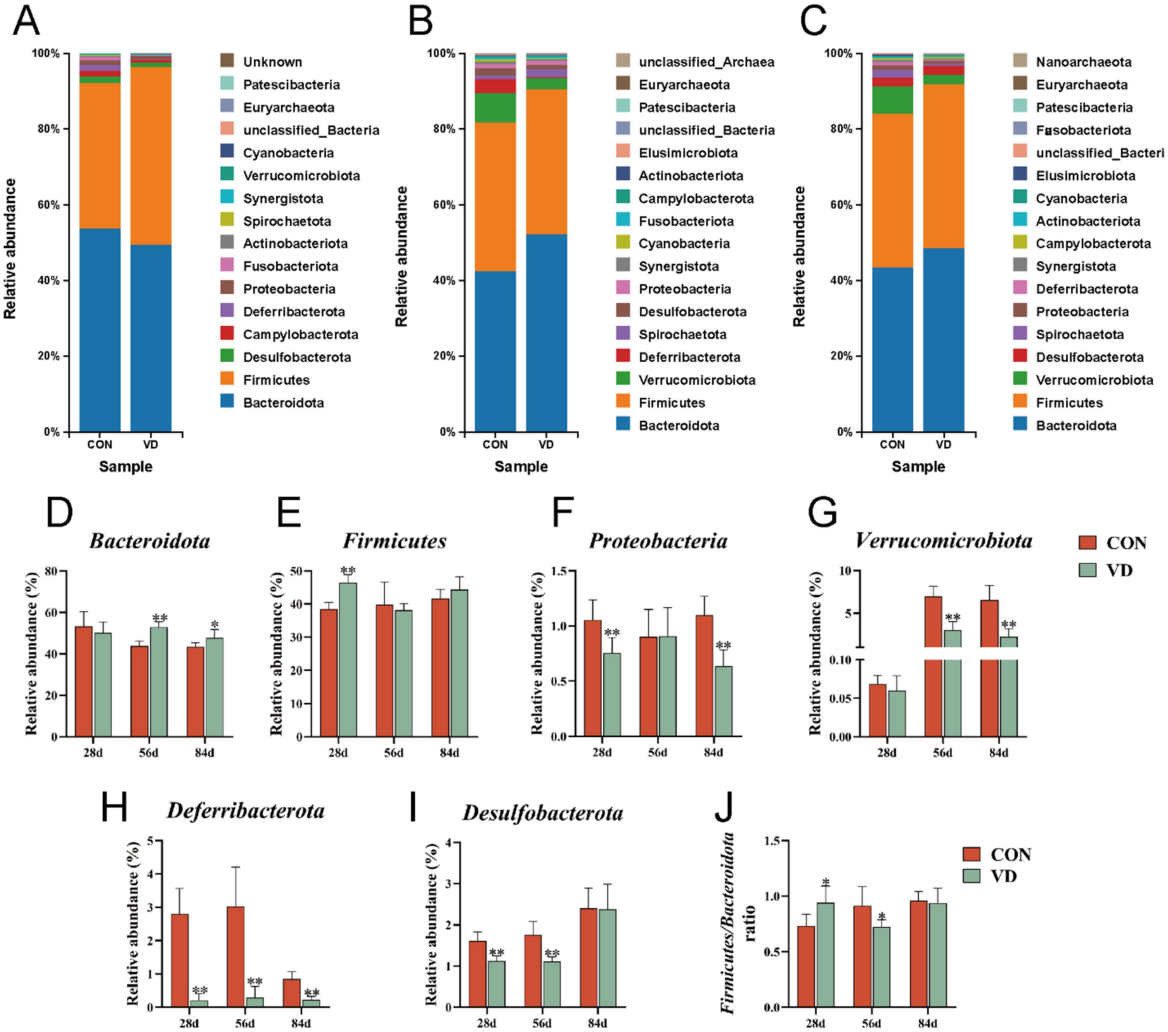
The relative abundance taxa of cecal microbiota in broilers at the phylum level. **(A–C)** Relative abundance taxa at 28, 56 and 84 days of age. **(D–I)** The relative abundance of *Bacteroidota*, *Firmicutes*, *Proteobacteria*, *Verrucomicrobiota*, *Deferribacterota* and *Desulfobacterota*, respectively. **(J)** The ratio of *Firmicutes*/ *Bacteroidota*. In panels **(D–J)**, data are presented as mean ± SEM (*n* = 6). *p < 0.05; **p < 0.01.

Based on the results of OUT delineation and taxonomic status identification, the specific composition of each sample at each taxonomic level could be obtained. At the phylum level, a total of 19 microbial phyla were identified in the cecal contents of broilers from the two groups (Figures 5A–C). We selected the dominant bacterial phyla that exhibited relatively high abundance across all three growth stages of broilers for analysis, resulting in the identification of six phyla: *Bacteroidota*, *Firmicutes*, *Proteobacteria*, *Verrucomicrobiota*, *Deferribacterota*, and *Desulfobacterota* (Figures 5D–I).

At 28 days of age, compared to the CON group, supplementary VD_3_ increased significantly (*p* < 0.05) the relative abundance of *Firmicutes* (38.49% vs. 46.92%), while reduced (*p* < 0.05) that of *Proteobacteria* (1.04% vs. 0.74%), *Deferribacterota* (1.70% vs. 0.24%), and *Desulfobacterota* (1.72% vs. 1.21%). Additionally, the ratio of the *Firmicutes* / *Bacteroidota* (F/B; 0.72 vs. 0.95) was significantly increased (*p* < 0.05; Figure 5J), while there was no significant change in the relative abundance of *Bacteroidota* (53.75% vs. 49.53%). At 56 day of age, supplementary VD_3_ increased significantly (*p* < 0.01) the relative abundance of *Bacteroidota* (42.41% vs. 52.33%), and reduced (*p* < 0.01) that of *Deferribacterta* (3.74% vs. 0.33%), *Desulfobacterota* (1.90% vs. 1.15%), and *Verrucomicrobiota* (7.74% vs. 2.84%). The ratio of the F / B (0.93 vs. 0.73) was significantly decreased (*p* < 0.05), while there was no significant change in the relative abundance of *Firmicutes* (39.33% vs. 38.27%). At 84 days of age, supplementary VD_3_ increased significantly (*p* < 0.05) the relative abundance of *Bacteroidota* (43.49% vs. 48.62%), and reduced (*p* < 0.01) that of *Proteobacteria* (1.15% vs. 0.72%), *Deferribacterota* (0.95% vs. 0.27%), and *Verrucomicrobiota* (7.18% vs. 2.46%). However, there were no significant changes in the F / B ratio. Obviously, the VD_3_ supplementation had a notable impact on the cecal microbiota composition at the phylum level in broilers.

#### 3.5.3 Variation in cecal microbiota composition at the genus and species levels

**Figure 6:**
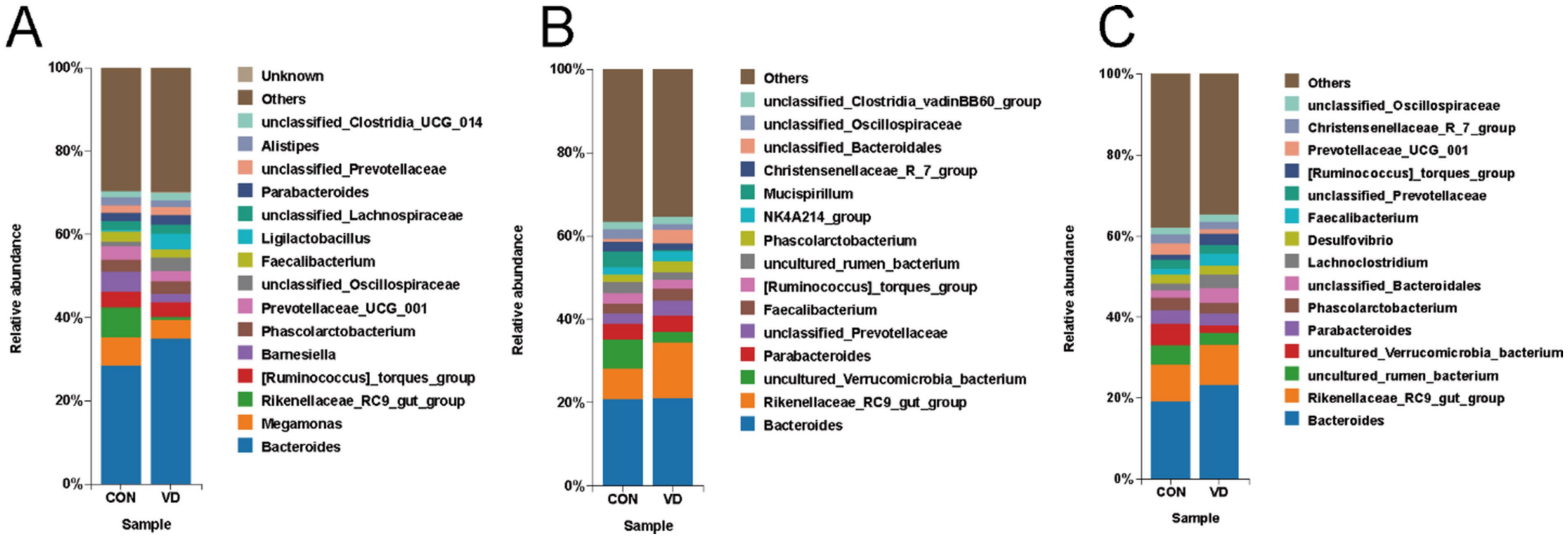
The relative abundance of cecal microbiota at the genus level. **(A–C)** Relative abundance taxa at 28, 56, and 84 days of age. The top 15 genera by relative abundance were shown, while the remaining genera were categorized as “Others”.

The top 15 genera by relative abundance in the cecal microbiota were presented in Figure 6. At 28 days of age, the most dominant bacterial genera in the CON and VD groups were *Bacteroides*, *Megamonas*, *Rikenellaceae_RC9_gut_group*, *[Ruminococcus]_torques_group* and *Barnesiella*. The relative abundance of *Bacteroides* significantly increased (*p* < 0.05), while that of *Megamonas*, *Rikenellaceae_RC9_gut_group* and *Barnesiella* significantly decreased (*p* < 0.05) in the VD group (Supplementary Table S1). At 56 days of age, the most dominant genera were *Bacteroides*, *Rikenellaceae_RC9_gut_group*, *uncultured_Verrucomicrobia_bacterium*, *Parabacteroides*, and *unclassified_Prevotellaceae* in the CON and VD groups. The relative abundance of *Rikenellaceae_RC9_gut_group* and *unclassified_Prevotellaceae* significantly increased (*p* < 0.05), while that of *uncultured_Verrucomicrobia_bacterium* significantly decreased (*p* < 0.05) in the VD group (Supplementary Table S2). At 84 days of age, the most dominant genera were *Bacteroides*, *Rikenellaceae_RC9_gut_group*, *uncultured_rumen_bacterium*, *uncultured_ Verrucomicrobia_bacterium*, and *Parabacteroides*. The relative abundance of *Bacteroides* and *Rikenellaceae_RC9_gut_group* significantly increased (*p* < 0.05), while that of *uncultured_rumen_bacterium* and *uncultured_Verrucomicrobia_bacterium* reduced significantly (*p* < 0.05) in the VD group (Supplementary Table S3). Moreover, supplementary VD_3_ also enhanced (*p* < 0.05) the relative abundance of *Ligilactobacillus* at day 28, *Faecalibacterium* and *Phascolarctobacterium* at day 56, and *Faecalibacterium* and *Lachnoclostridium* at day 84.

**Figure 7:**
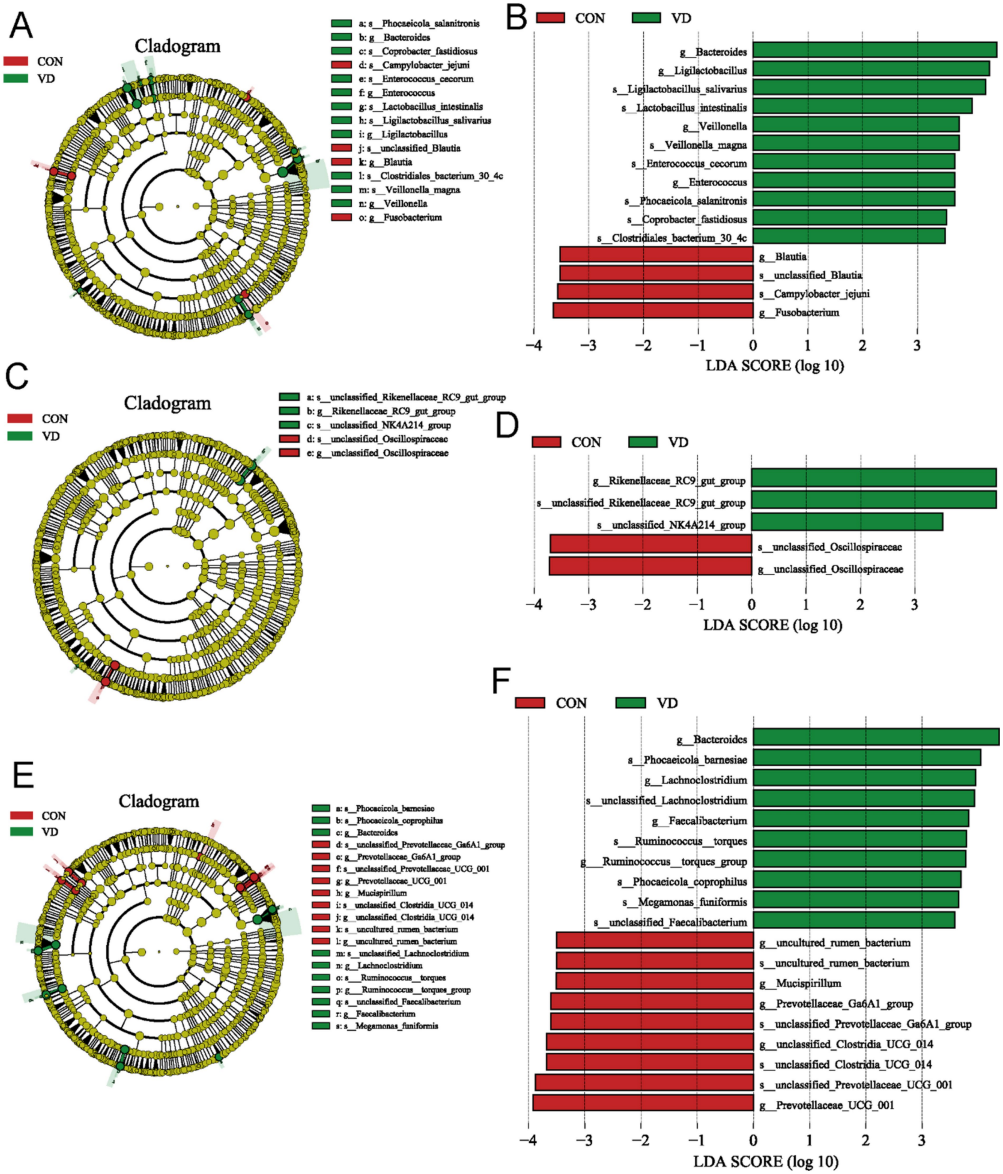
LEfSe taxonomic cladogram and LDA score of cecal microbiota. **(A,B)** LEfSe taxonomic cladogram and LDA score at 28 days of age. **(C,D)** LEfSe taxonomic cladogram and LDA score at 56 days of age. **(E,F)** LEfSe taxonomic cladogram and LDA score at 84 days of age. The circles radiating from the center to the outer edges of the evolutionary branch map represent classification levels from phylum to species, with the circle’s diameter proportional to the taxon’s abundance. The yellow nodes represent taxonomic units that exhibit no significant differences between groups. The LDA histograms display the LDA scores, and the difference is significant when LDA >3.5. g, genus; s, species.

The Linear Discriminant Analysis (LDA) combined with LDA Effect Size (LEfSe) analysis was employed to further examine the changes in the cecum microbiota at both the genus and species levels in broilers (LDA scores >3.5). At 28 days of age, there were 11 biomarkers enriched in the VD group, such as *g_Bacteroides*, *g_Ligilactobacillus*, *s_Ligilactobacillus_salivarius*, *s_Lactobacillus_intestinalis*, and *g_Veillonella*, and 4 biomarkers enriched in CON group, such as *g_Blautia*, and *g_ Fusobacterium* (Figures 7A,B). At 56 days of age, *g_Rikenellaceae_RC9_gut_group* was identified as biomarker in the VD group (Figures 7C,D). At 84 days of age, there were 10 biomarkers in the VD group, including *g_Bacteroides*, *g_Lachnoclostridium*, *g_Faecalibacterium* and *g_Ruminococcus_torques_group*, and the CON group was enriched with *g_Prevotellaceae_UCG_001*, *g_Prevotellaceae_Ga6A1_group*, *g_Mucispirillum* (Figures 7E,F).

#### 3.5.4 Correlation of microbiota with indicators of lipid metabolism

**Figure 8:**
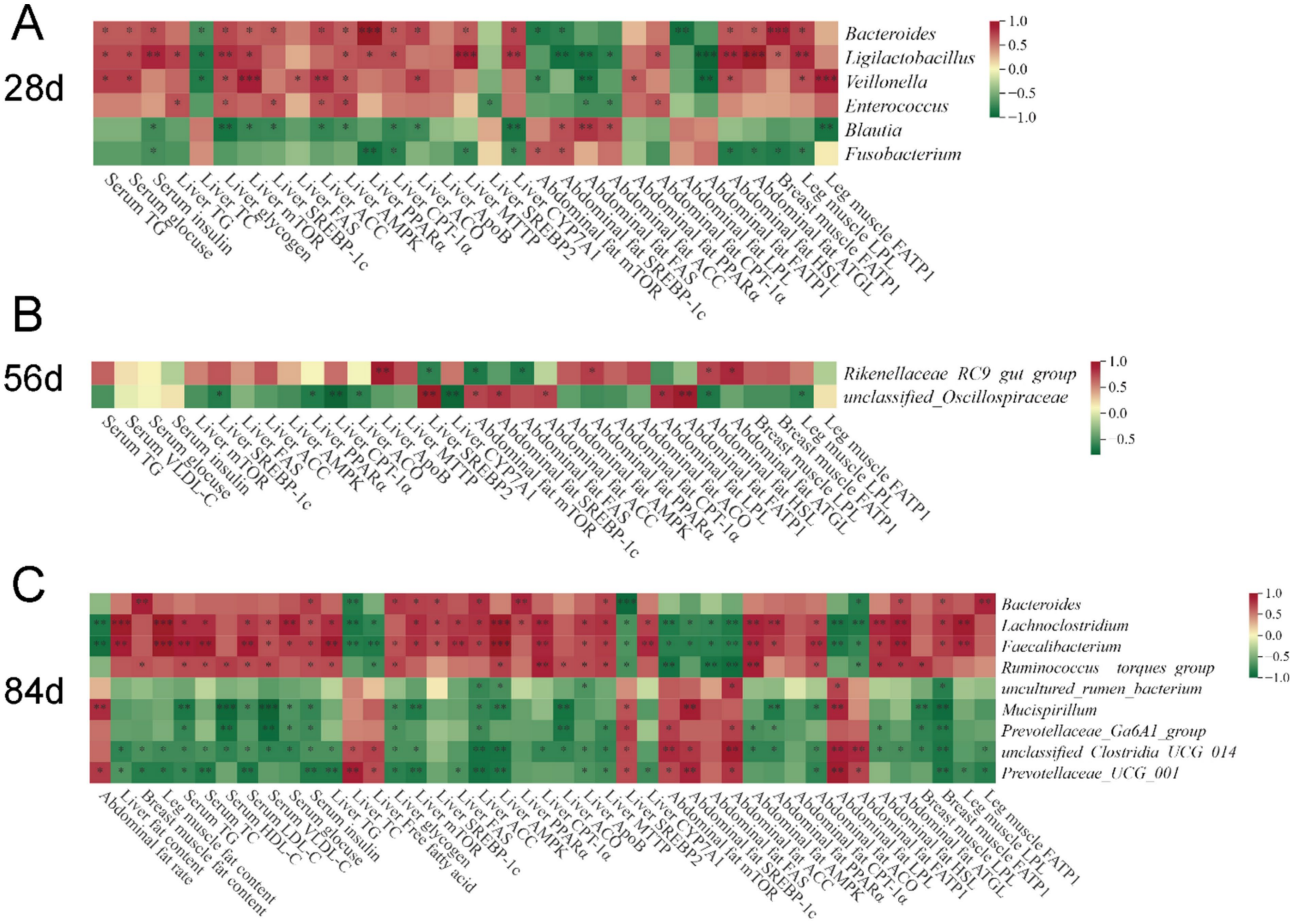
Correlation heatmap of cecal biomarker bacteria and differential lipid metabolism indicators of broilers. **(A)** 28 days of age; **(B)** 56 days of age; **(C)** 84 days of age. Spearman’s correlations were calculated between cecal biomarker bacterial genera and all significantly different fat content, gene expression in tissues, and serum parameters. Colors of squares represent r values of Spearman’s correlation coefficient, with red indicating a positive correlation and green signifying a negative correlation. *p < 0.05, **p < 0.01, ***p < 0.001.

To investigate the relationships between intestinal microbiota and lipid metabolism in broilers, we performed a Spearman correlation analysis (Figure 8) based on the aforementioned cecal biomarker microbiota at the genus level, lipid metabolism indicators, and gene expression data to determine the microbes linked to the regulatory effects of VD_3_ on lipid metabolism. At 28 days of age, the content of TG, glucose, and insulin in the serum, glycogen content and the expression of the mTORC1/SREBP-1c pathway (*mTOR*, *SREBP-1c*, and *ACC*), AMPK/PPARα/CPT1 pathway (*AMPK*, *PPARα*, *CPT-1α*, and *ACO*), *MTTP* and *CYP7A1* in the liver, as well as the expression of lipolytic pathway (*HSL* and *ATGL*) in the abdominal adipose tissue, and *FATP1* in breast muscle, *LPL* in leg muscle were positively correlated with the relative abundance of *Bacteroides*, *Ligilactobacillus* and *Veillonella*. Moreover, the liver TC content, and the expression of *mTOR*, *SREBP-1c* and *FATP1* in the abdominal adipose tissue were negatively correlated with *Bacteroides* and *Ligilactobacillus* (Figure 8A).

At 56 days of age, *Rikenellaceae_RC9_gut_group* was positively correlated with the expression of *ApoB* in the liver, as well as *PPARα*, *HSL* and *ATGL* in abdominal adipose tissue, exhibiting a negative correlation with the expression of *SREBP2* in the liver, *mTOR* and *FAS* in the abdominal adipose tissue (Figure 8B).

At 84 days of age, *Lachnoclostridium* and *Faecalibacterium* exhibited a positive correlation with the fat content in the liver and leg muscle, serum metabolism parameters (TG, TC, LDL-C, VLDL-C, glucose, and insulin), liver TG, the expression of mTORC1/SREBP-1c pathway (*mTOR*, *SREBP-1c*, *FAS*, and *ACC*) and AMPK/PPARα/CPT1 pathway (*AMPK*, *PPARα*, and *CPT1α*), VLDL assembly (*ApoB* and *MTTP*), and *CYP7A1* in liver, and AMPK/PPARα/CPT1 pathway (*AMPK*, *PPARα*, and *ACO*), lipolytic pathway (*HSL* and *ATGL*) in the abdominal adipose tissue, and extrahepatic lipid uptake (*FATP1* in breast muscle and *LPL* in leg muscle), while exhibiting a negative correlation with other indicators, such as the abdominal fat rate, the TC and FFAs content and the expression of *SREBP2* in the liver, and the expression of mTORC1/SREBP-1c pathway (*mTOR*, *SREBP-1c*, *FAS*, and *ACC*), *LPL*, and *FATP1* in the abdominal adipose tissue (Figure 8C). However, contrasting results were observed for *Prevotellaceae_UCG_001* in comparison to these two genera. Additionally, *Bacteroides* was positively correlated with fat content in breast muscle, serum insulin and liver glycogen content, the expression of *mTOR*, *SREBP-1c*, *ACC*, *PPARα* and *MTTP* in the liver, and *ATGL* in abdominal adipose tissue, and *FATP1* in breast and leg muscle, while showing a negative correlation with liver TC, the expression of *SREBP2* in the liver, and *FATP1* in abdominal adipose tissue. However, the opposite result to *Bacteroides* was observed for *Prevotellaceae_UCG_001*.

#### 3.5.5 Functional projections

**Figure 9:**
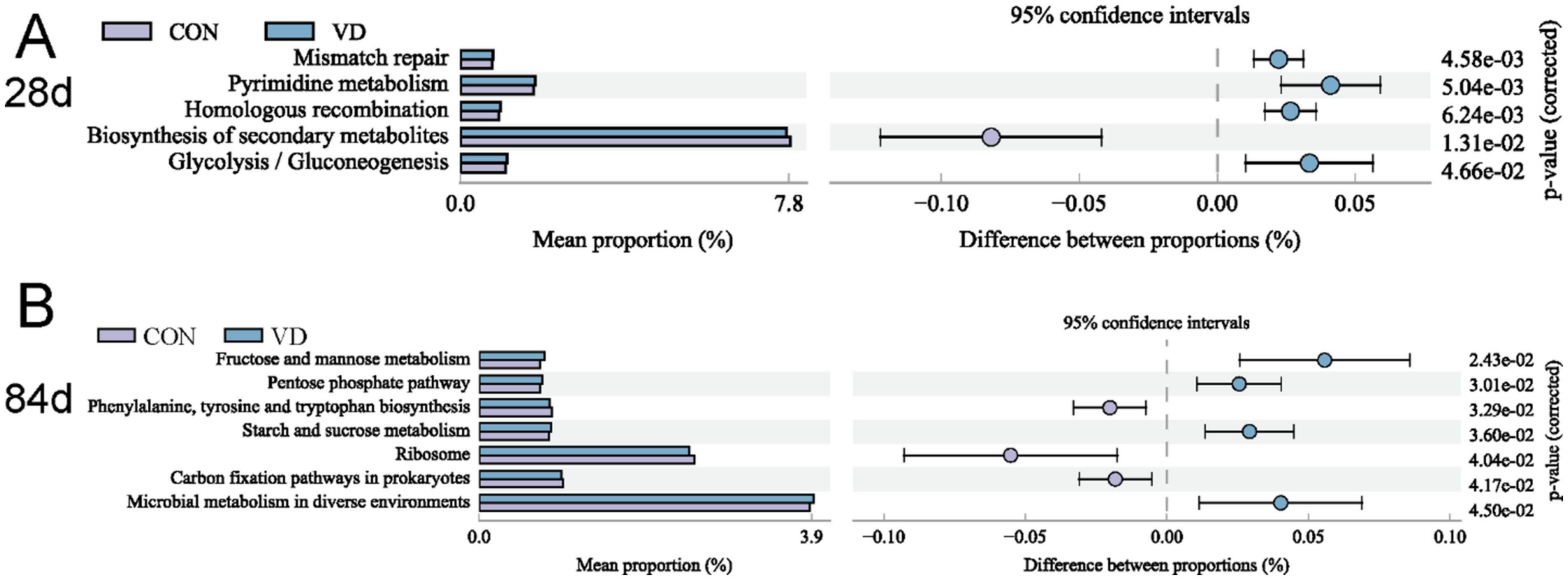
Differential KEGG level 3 metabolic pathways between groups. **(A)** 28 days of age; **(B)** 84 days of age. The *p*-value on the right is derived from G-test in the statistical analysis of taxonomic and functional profiles (STAMP) software, where *p*-value <0.05 indicates a significant difference.

To gain deeper insights into the variations in function of cecal microbiota between the CON and VD group, we performed KEGG enrichment prediction analysis on 16S rDNA sequencing data using the Integrated Microbial Genomes (IMG) database^2^ by PICRUST2. At 28 days of age, the relative abundance of pathways related to cellular processes (such as mismatch repair, pyrimidine metabolism and homologous recombination) and glycolysis / gluconeogenesis was significantly higher, while there was a decrease (*p* < 0.05) in the abundance of secondary metabolite biosynthesis in the VD group (Figure 9A). At 56 days of age, although several glucose metabolism and amino acid biosynthesis pathways were enriched, none were statistically significant (*p* > 0.05) between groups (Supplementary Figure S1). At 84 days of age, the relative abundance of energy metabolic pathways, such as fructose and mannose metabolism, pentose phosphate pathway, starch and sucrose metabolism, were significantly increased, while that of phenylalanine, tyrosine and tryptophan biosynthesis was decreased (*p* < 0.05) in the VD group (Figure 9B).

## 4 Discussion

### 4.1 Growth performance and carcass traits

In contemporary broiler production, excessive accumulation of abdominal fat is a significant factor influencing the carcass quality and feed conversion of broiler chickens. Increasing evidences have shown that VD_3_ regulated lipid metabolism in humans and animals (12, 28), and that there was a significant association between VD_3_ levels, gut microbiota and lipid metabolism (18–20). However, the effect of VD_3_ on fat accumulation and lipid metabolism in broilers has been less frequently reported. In this study, supplementation with VD_3_ increased ADG of broilers aged 57–84 and 1–84 days, and the final body weight and leg muscle rate at 84 days of age. However, the ADFI during each growth period was not significantly influenced by VD_3_ supplementation, resulting in a non-significant FCR. Additionally, the supplementary VD_3_ significantly reduced abdominal fat rate and increased the fat content in liver, leg and breast muscle of broilers at 84 days of age, while having no effects on those of broilers at 28 and 56 d of age. Consistent with this, it was reported that VD_3_ promoted growth performance and bone development by exerting a series of regulatory effects in broilers (24, 29). Considering that serum VD_3_ levels were continuously elevated by supplemental VD_3_, we proposed that the VD_3_ added to the basal diet enhanced growth performance, as well as improved carcass quality by differentially influencing fat accumulation in various tissues of broilers, with these effects mainly occurring during the finishing period.

Additionally, it should be noted that VD_3_ stored in edible parts, such as muscle and adipose tissue, did not present food safety issues under the supplementary dosage of 3,750 IU/kg of VD_3_ in this study. VD_3_ mainly store in fat-rich tissues and organs due to its fat-soluble property. The liver and adipose tissue of humans and animals expressed and secreted 24-hydroxylase CYP24A1 (30). CYP24A1 catalyzed the breakdown of 25(OH)D_3_ and 1,25(OH)_2_D_3_ into calcitroic acid and other inactive metabolites, and 1,25(OH)_2_D_3_ induced CYP24A1, promoting its own degradation and ultimately self-regulating through inactivation pathways to maintain homeostasis (31). Morrisey et al. (32) evaluated VD_3_ toxicity through renal histopathological examination, a sensitive method for detecting VD toxicity, and showed that a dose of 400,000 IU/kg VD_3_ resulted in calcification of renal tubules in chickens, whereas 40,000 IU/kg VD_3_ exhibited no toxicity, which was determined to be the maximum food safety level. Additionally, a VD_3_ addition level of 12,000 IU/kg showed no adverse effects on laying hens and their eggs (33).

### 4.2 Lipid metabolism in the liver

The lipid metabolism in the animals is in dynamic equilibrium, with lipid levels in the liver, peripheral tissues, and blood being interconnected and mutually affecting each other (34). Liver lipid levels served as a crucial indicator of the overall lipid metabolism in broilers (35). The supplementary VD_3_ increased the liver TG and glycogen levels, as well as the liver organ index, while reducing TC levels in broilers at 28 and 84 days of age, along with a decrease in FFA levels at 84 days of age. The increased relative liver weight indicated a rise in lipid content rather than liver enlargement, as it has been established that VD_3_ doses below 40,000 IU/kg are non-toxic to broiler chickens (32).

Poultry primarily synthesize lipids in the liver, and substantial evidence has shown that the level of liver lipid metabolism depends on *de novo* lipogenesis (DNL), β-oxidation, and VLDL export (36, 37). To gain a deeper understanding of the effect of VD_3_ on liver lipid metabolism, we analyzed the expression of genes and the activity of enzymes related to lipid metabolism. The supplementary VD_3_ significantly up-regulated the expression of *mTOR*, *SREBP-1c*, *FAS* and *ACC*, and increased the activity of FAS and ACC enzymes in the liver. In the same way, the expression of *AMPK*, *PPARα*, *CPT-1α* and *ACO* and the activity of CPT-1α enzyme in the liver were significantly increased by the supplementary VD_3_. The mTORC1/SREBP-1c pathway was closely related to fat accumulation in animals (38). The transcription factor SREBP-1c regulated glycolysis and lipogenesis by modulating the expression of its target genes, such *ACC* and *FAS* (39, 40). The mTOR pathway was activated by signals like insulin, which led to the process of SREBP-1c from its precursor to a mature protein and its translocation to the nucleus, thereby promoting the transcription of lipogenic genes (39). PPARα, a type of nuclear receptor, is activated by free fatty acids and various lipid molecules, stimulating to the transcription of genes involved in fatty acid transport, β-oxidation, and lipoprotein metabolism (41). AMPK activated PPARα signaling pathways, promoting fatty acid oxidation (42). AMPK activation enhanced lipid metabolism by switching from carbohydrate metabolism to lipid oxidation, leading to a reduction of both liver TG and obesity in mice (43). Both *CPT-1α* and *ACO*, as the target genes of PPARα, were involved in β-oxidation of fatty acids. CPT-1α was the rate-limiting enzyme for fatty acid oxidation and served as the delivery of long-chain fatty acids from the cytoplasm into the mitochondria (44, 45). It has been demonstrated that the activation of the AMPK/PPARα/CPT1 pathway stimulated the lipid metabolism and reduced lipid accumulation in obese mice (46). Additionally, the supplementation with VD_3_ reduced the expression of *SREBP2* and increased the expression of *CYP7A1* in the liver. The transcription factor SREBP2 stimulated cholesterol biosynthesis, while the CYP7A1 enzyme was responsible for converting cholesterol into bile acids in the liver of poultry (47). Therefore, it is reasonable to assume that supplementation with VD_3_ could enhance glucose metabolism, DNL, and lipid oxidation, all of which contributed to an increase in TG and glycogen levels in the liver of broilers at 28 and 84 days of age.

This was also supported by elevated insulin concentration, and reduced the level of FFAs and the expression of lipolytic gene *HSL* and *ATGL* in liver. The main function of insulin in hepatic lipid metabolism is to regulate lipid storage by exerting control over DNL, FFA oxidation and VLDL export (48). Hormone-sensitive lipase (HSL) and adipose triglyceride lipase (ATGL) are key enzymes in the lipolytic pathway, hydrolyzing stored triglycerides to release FFAs for β-oxidation (49, 50). Insulin profoundly inhibited lipolysis primarily by suppressing the activity of HSL and stimulated glycogen accumulation through a coordinated increase in glucose transport and glycogen synthesis (51).

Conversely, VD_3_ could decrease TC levels by inhibiting cholesterol synthesis and promoting the conversion of cholesterol to bile acid efflux in the liver. However, the alterations in gene expression and enzyme activity related to lipid metabolism in the liver of broilers at 56 days of age did not result in significant changes in TG content, which could be attributed to the fact that this stage is in a period of rapid growth and requires increased energy to support growth.

### 4.3 Lipid transport in the blood

The lipids produced in the liver were transported by the bloodstream to extrahepatic tissues for storage or oxidation. Serum TG and TC levels are important indicators of lipid transport in the body. HDL, LDL and VLDL are all types of lipoproteins responsible for transporting lipids through the bloodstream, which can provide an insight into overall lipid metabolism. HDL is involved in cholesterol removal and transport triglycerides back to the liver, LDL is responsible for delivering cholesterol to cells, and VLDL transports triglycerides from the liver to other tissues (52). Lipoproteins are composed of a lipid core encased in a layer of phospholipids and apolipoproteins, with the liver serving as a crucial site for the synthesis of apolipoproteins. Both Apolipoprotein B (ApoB) and Microsomal Triglyceride Transfer Protein (MTTP) are critical components involved in the assembly of lipoproteins (53). In this study, the supplementary VD_3_ elevated serum TG and VLDL levels, indicating that it could enhance the transport of triglycerides to peripheral tissues. Additionally, the elevated serum HDL levels at 84 days of age indicated an increase in lipid transport back to the liver, which contributed to a rise in liver TG levels, attributed to the rise in body fat mass during the later stage of growth. These results were also supported by the expression of *ApoB* and *MTTP* in the liver, which was elevated by the supplementary VD_3_ in this study.

### 4.4 Lipid metabolism in the adipose tissue and muscle

The accumulation of triglycerides within lipid droplets was one of the causes of adipose tissue expansion, which was positively correlated with abdominal fat mass (54). The sources of fat in animal tissues include cellular uptake and DNL. DNL in the liver supplied the necessary fatty acids to other tissues by cellular uptake (55). However, the adipose tissue was also an important site for DNL in broilers (56). Fatty acids for cellular uptake are derived from the hydrolysis of blood triglycerides mediated by lipoprotein lipase (LPL) (54). Triglycerides produced in the liver were transported by apolipoproteins through the bloodstream to extrahepatic tissues (such as adipose tissue and muscle) (57). LPL catalyzed the hydrolysis of the triglycerides in VLDL to release FFAs, and promoted the uptake of fatty acids by tissues (54). FATP1 promoted cellular fatty acid uptake and adipocyte hypertrophy by mediating the synthesis of fatty acids into triglycerides (58). The supplementary VD_3_ suppressed the expression of mTOR/SREBP-1c pathway related genes (*mTOR*, *SREBP-1c*, *FAS* and *ACC*), while increased the expression of AMPK/PPARα/CPT-1α pathway related genes (*AMPK*, *PPARα*, *CPT-1α* and *ACO*) and lipolytic enzyme-encoding genes (*HSL* and *ATGL*), suggesting that VD_3_ could inhibit lipogenesis, and promote lipolysis and fatty acid β-oxidation in abdominal adipose tissue. Moreover, we found also that VD_3_ significantly downregulated the expression of *LPL* and *FATP1*, and decreased the activity of LPL enzyme in abdominal adipose tissue of broilers at 84 days of age, suggesting that VD_3_ suppressed the uptake of lipids transported from the liver by abdominal adipocytes in broilers. Consequently, the supplementary VD_3_ decreased the abdominal fat rate by inhibiting DNL and cellular uptake pathways while enhancing the lipolysis and β-oxidation pathway.

Unlike abdominal fat reduction, the supplementary VD_3_ significantly increased fat content in breast and leg muscles. Elevated liver TG levels, blood lipid transport, and the expression of *LPL* and *FATP1* in breast and leg muscles due to VD_3_ could result in an increased cellular lipid uptake, which partially accounted for the rise in muscle fat content. However, muscle tissue mainly consists of myocytes, adipocytes, and connective tissue cells. Unlike abdominal fat, the formation of IMF is more complex due to the interference of myocytes (56). Thus, our experimental evidence was insufficient to fully clarify the promotion of muscle fat deposition by supplementary VD_3_ in broilers.

The supplementary VD_3_ differentially influenced lipid metabolism and fat deposition in the liver, muscle and adipose tissue of broilers, which could reflect tissue-specific effects of VD_3_. As previously discussed, VD_3_ enhanced both the mTORC1/SREBP-1c and AMPK/PPAR*α*/CPT-1α signaling pathways while decreasing the expression of lipolytic genes in the liver. Conversely, in adipose tissue, the expression of the mTOR/SREBP-1c pathway was suppressed, whereas the expression of the AMPK/PPARα/CPT-1α pathway and lipolytic genes was elevated by supplementary VD_3_. Researches have confirmed that nutrients affect lipid metabolism and fat distribution by influencing the expression of related genes in the liver, muscle, and adipose tissues of animals in a tissue-specific manner (59, 60). VD_3_ could regulate lipid metabolism through multiple pathways, and its genomic and epigenetic effects that depend on VDR, as well as its non-genomic effects, have been reviewed in detail (61). The finding of VD_3_ that elevated insulin levels in this study could provide anther cue for understanding the regulation of VD on lipid metabolism, but further research is needed.

### 4.5 Variation in cecal microbiota composition and its effect on lipid metabolism

VD_3_ supplementation significantly altered α-diversity of cecal microbiota. We also observed that VD_3_ significantly affected the structure of cecal microbiota in broilers, especially in the grower and finisher stage. PICRUSt2 functional prediction for cecal microbiota in broilers at 28 days of age indicated that VD_3_ increased the abundance of metabolic pathways involved in the cellular processes and energy metabolism. The age of 1–28 days (early growth stage) is a critical period for shaping gut microbiome composition, and the enhancement of VD_3_ on the cellular processes could contribute to a healthier microbial population, reducing the risk of mutations and promoting overall microbiome stability. For broilers at 84 days of age, VD_3_ increased the abundance of energy metabolic pathways, while decreasing the biosynthesis of amino acids, all of which contributed an increased capacity of the microbiota to utilize energy substances. This indicated that VD_3_ could improve the availability of nutrients and energy storage by modulating the metabolic activities of the cecal microbiota in the late growth stage of broilers.

The cecal microbiota significantly contributed to fat deposition and could independently account for 21% of the variance in the abdominal fat mass after correcting for host genetic effects (62), and the enterohepatic axis had a significant effect on lipid metabolism in poultry (63). Previous study found that VD_3_ reduced the expression of lipogenic genes in the liver by regulating the gut microbiota in mice (64). Here, we primarily discussed the potential effects of VD_3_ on lipid metabolism by influencing cecal microbiota composition in broilers.

The composition of cecal microbiota at the phyla varied significantly across different growth stages in broilers, but *Firmicutes* and *Bacteroidetes* were always most predominant, which was consistent with previous study on broilers (26). The relative abundance of *Firmicutes* at 28 days of age and that of *Bacteroidota* at 56 and 84 days of age were increased by the supplementary VD_3_, while showing inconsistent changes in F/B ratio. *Firmicutes* and *Bacteroidetes* in the gut collectively influenced the host’s energy absorption and storage, and the F/B ratio affected the host’s ability to obtain energy from feed (65). Both *Firmicutes* and *Bacteroidetes* promoted fat deposition in animals, and *Firmicutes* exhibited a stronger promotion compared to *Bacteroidetes* (66). Nakamoto et al. (67) found that both *Firmicutes* and *Bacteroidetes* were associated with liver lipid metabolism and stimulated the expression of lipogenic enzyme-encoding gene such as *FAS*, stearoyl-CoA desaturase-1 (*SCD1*), and diacylglycerol O-acyltransferase 2 (*DGAT2*) in mice. An increase in *Firmicutes* enhanced carbohydrate metabolism and energy absorption (68), while *Bacteroidetes* fermented indigestible polysaccharides in the gut, providing energy to the host (69). Gut *Firmicutes* and *Bacteroidetes* have been shown to influence lipid metabolism and obesity in animals by producing SCFAs (70). Additionally, we also observed that VD_3_ reduced the relative abundance of these results suggested that the supplementary VD_3_ could enhance energy utilization and lipogenesis in broilers by affecting the composition of gut microbiota.

Based on the analysis of cecal microbial composition at the genus levels, we found that *Bacteroides*, *Rikenellaceae_RC9_gut_group* and *Prevotellaceae_UCG_001* were all dominant bacterial genera in broilers at three different ages, all of which belong to the *Bacteroidetes*. VD_3_ significantly increased the abundance of *Bacteroides* in the cecum of broilers at 28 and 84 d of age, and LEfSe analysis also showed that *Bacteroides* was a biomarker for the VD group. *Bacteroides* species utilized various plant polysaccharides and host-derived glycans to produce beneficial end products, fostering the mutualistic relationship between humans and their resident intestinal *Bacteroides* (71). *Bacteroides* supplementation improved glucose tolerance and intestinal barrier structure, and reduced the adiposity in female mice fed a high-fat diet (HFD) (72). Moreover, *Bacteroides* reduced body weight gain, and cholesterol and triglyceride levels in the liver and serum in HFD-fed mice (73). In this study, we found that *Bacteroides* was positively correlated with glycogen content and the expression of the mTOR/SREBP-1c and AMPK/PPARα/CPT1 pathways in the liver of 28- and 84-day-old broilers. However, *Bacteroides* was negatively correlated with the mTOR/SREBP-1c pathway, while showing a positive correlation with the lipolysis pathway in the abdominal adipose tissue of 28-day-old broilers.

*Rikenellaceae_RC9_gut_group* produced SCFAs and was correlated with a lot of lipid metabolism pathways (74). It was found that the abundance of *Rikenellaceae_RC9_gut_group* was low in obese mice and negatively correlated with serum glucose, TG, TC, LDL-C, insulin, and hepatic TC (75), and positively correlated with fecal butyrate levels (76). In contrast, the reduction of *Rikenellaceae_RC9_gut_group* in the gut was accompanied by a decrease in serum levels of TC, TG, and LDL-C in weaned piglets (77). In this study, the supplementary VD_3_ significantly increased the abundance of *Rikenellaceae_RC9_gut_group* at 56 and 84 days of age, while decreasing it at 28 days of age, suggesting that this genus could be relevant one in VD_3_ regulation. Furthermore, *Rikenellaceae_RC9_gut_group,* identified as a marker genus of VD group at 56 days of age, showed a positive correlation with the expression of *PPARα*, *HSL* and *ATGL,* while exhibiting a negative correlation with the expression of *mTOR* and *FAS* in the abdominal adipose tissue.

*Prevotellaceae_UCG_001*, a member of the family *Prevotellaceae*, generated enzymes that degrade cellulose and xylan to produce SCFAs, which play a protective role against gut inflammation (78). The abundance of *Prevotellaceae_UCG_001* was positively correlated with the AMPK signaling pathway that promoted glycolysis and fatty acid oxidation in mice (79). In this study, the abundance of *Prevotellaceae_UCG_001* in broilers at 84 days of age was reduced by the supplementary VD_3_, and its correlation with lipid metabolism pathways was contrary to that of *Bacteroides*, except for a positive correlation with the abdominal fat rate. *Bacteroides* and *Prevotellaceae* have been reported to exhibit mutual antagonism or competition in the distal gut (69). The decrease in the abundance of *Prevotellaceae_UCG_001* might be related to the increase in the abundance of *Bacteroides* by supplementation with VD_3_ in this study. These results indicated that the regulation of VD_3_ on lipid metabolism, including the enhanced lipid metabolism in the liver, and the reduced lipogenesis and the enhanced lipolysis and lipid oxidation in adipose tissue, was linked to an increased abundance of *Bacteroides* and *Rikenellaceae_RC9_gut_group*, along with a decreased abundance of *Prevotellaceae_UCG_001* in the cecum of broilers.

In this study, we also observed that VD_3_ increased the abundance of *Lachnoclostridium* at 84 days of age and that of *Faecalibacterium* at 56 and 84 days of age. These two bacterial genera belong to the phylum *Firmicutes* and contribute to the production of acetate and butyrate (80), which serve as energy sources for gut epithelial cells, and influence host’s lipid metabolism by impacting glucagon-like peptide-1 (GLP-1) and peptide YY (PYY). Although SCFAs are not considered a major energy source for broilers, current evidence suggested that gut microbiota, including *Faecalibacterium* and *Lachnoclostridium*, affected overall lipid metabolism through the gut-brain axis in humans (81). SCFAs affected adipose tissue biology and physiology, potentially influencing lipid metabolism (82). Studies have demonstrated that the acetate inhibited body fat accumulation and hepatic lipid metabolism in mice by enhancing the expression of *PPARα*, *CPT-1* and *ACO* in the liver (83). An increased abundance of gut *Lachnoclostridium* was associated with enhanced SCFAs production and reduced blood glucose levels in rats (84). However, Nogal et al. (85) reported that a higher abundance of *Lachnoclostridium* decreased circulating acetate levels, resulting in an increase in visceral fat in humans. Additionally, *Faecalibacterium prausnitzii*, the most recognized species in *Faecalibacterium*, enhanced fatty acid oxidation and adiponectin signaling in the liver and visceral adipose tissue, while reducing adipocyte size in HFD-fed mice (86). The correlation analysis revealed that both *Lachnoclostridium* and *Faecalibacterium* were positively correlated with the fat and TG content, the mTOR/SREBP-1c and the AMPK/PPARα/CPT1 pathway in the liver, as well as serum parameters related to lipid metabolism and VLDL assembly. However, they exhibited a negative correlation with abdominal fat rate, mTOR/SREBP-1c pathway, and cellular lipid uptake in abdominal adipose tissue, while showing a positive correlation with lipolysis and AMPK/PPARα/CPT1 pathway. Conversely, these two bacterial genera were positively correlated with the fat content and cellular lipid uptake in breast and leg muscles. These results indicated that VD_3_ could differentially regulate lipid metabolism in different tissues by increasing the abundance of *Lachnoclostridium* and *Faecalibacterium* in the cecum of broilers, including that enhanced the lipid metabolism and transport in the liver and fat accumulation in muscles, as well as reduced fat deposition in adipose tissue by inhibiting DNL and lipid uptake and promoting lipolysis and fatty acid oxidation.

To sum up, VD_3_ could differentially regulate lipid metabolism and fat deposition in different tissues by influencing the composition of intestine microbiota in broilers. The increased abundance of *Bacteroides*, *Rikenellaceae_RC9_gut_group*, *Lachnoclostridium* and *Faecalibacterium* was linked to enhanced lipid metabolism and transport in the liver, as well as fat deposition in muscles. However, the increased abundance of *Bacteroides* and *Rikenellaceae_RC9_gut_group*, and the decreased abundance of *Prevotellaceae_UCG_001*, was associated with the reduced lipogenesis and enhanced lipolysis and lipid oxidation in adipose tissue.

## 5 Conclusion

In summary, the addition of VD_3_ on the basal diet enhanced growth performance, increased IMF in breast and leg muscles, and reduced the abdominal fat rate leading to an improvement in carcass quality by differentially regulating lipid metabolism in various tissues of broilers, with these effects primarily occurring during the finishing period. Moreover, the regulation of supplementary VD_3_ on lipid metabolism could was closely associated with an increased abundance of *Firmicutes* including *Lachnoclostridium and Faecalibacterium*, and *Bacteroidetes* including *Bacteroides* and *Rikenellaceae_RC9_gut_group*.

## Data Availability

The datasets presented in this study can be found in online repositories. The names of therepository/repositories and accession number(s) can be found at: https://www.ncbi.nlm.nih.gov/, PRJNA1195667.

## Footnotes

^1^ https://www.biocloud.net

^2^ https://img.jgi.doe.gov/cgi-bin/m/main.cgi/m/main.cgi
