# Supplementary material for: Effects of supplementation with vitamin D3 on growth performance, lipid metabolism and cecal microbiota in broiler chickens
Source: Front Vet Sci. 2025 Feb 6;12:1542637. doi: 10.3389/fvets.2025.1542637 (PMC11839666; doi:10.3389/fvets.2025.1542637)
Supplement: Supplementary file 1 [file Table_1.docx]

***Supplementary Material***

**Table S1.** Effect of dietary VD_3_ microbiota composition of the cecum at genus level of broilers at 28 days of age.

| **Item** | **CON group (%)** | **VD group (%)** | **SEM** | ***P*-Value** |
| --- | --- | --- | --- | --- |
| *Bacteroides* | 28.63 | 34.93^**^ | 1.44 | 0.009 |
| *Megamonas* | 6.78 | 4.51^*^ | 0.54 | 0.011 |
| *Rikenellaceae_RC9_gut_group* | 7.13 | 0.75^**^ | 0.43 | <0.001 |
| *[Ruminococcus]_torques_group* | 3.77 | 3.51 | 0.36 | 0.680 |
| *Barnesiella* | 4.82 | 2.12^**^ | 0.46 | 0.003 |
| *Phascolarctobacterium* | 2.81 | 3.02 | 0.43 | 0.984 |
| *Prevotellaceae_UCG_001* | 3.16 | 2.50 | 0.32 | 0.200 |
| *unclassified_Oscillospirac* | 1.21 | 3.17^**^ | 0.27 | <0.001 |
| *Faecalibacterium* | 2.37 | 1.95 | 0.39 | 0.474 |
| *Ligilactobacillus* | 0.47 | 3.83^**^ | 0.34 | <0.001 |

Note: Values with superscripts "*" indicate significant difference (*P*<0.05), "**" indicate significant difference (*P*<0.01).
