# Supplementary material for: Effects of supplementation with vitamin D3 on growth performance, lipid metabolism and cecal microbiota in broiler chickens
Source: Front Vet Sci. 2025 Feb 6;12:1542637. doi: 10.3389/fvets.2025.1542637 (PMC11839666; doi:10.3389/fvets.2025.1542637)
Supplement: Supplementary file 2 [file Table_2.docx]

***Supplementary Material***

**Table S2.** Effect of dietary VD_3_ microbiota composition of the cecum at genus level of broilers at 56 days of age.

| **Item** | **CON group (%)** | **VD group (%)** | **SEM** | ***P*-Value** |
| --- | --- | --- | --- | --- |
| *Bacteroides* | 20.87 | 21.07 | 1.10 | 0.873 |
| *Rikenellaceae_RC9_gut_group* | 7.40 | 13.49^**^ | 0.86 | 0.001 |
| *uncultured_Verrucomicrobia_bacterium* | 6.93 | 2.43^**^ | 0.34 | <0.001 |
| *Parabacteroides* | 3.85 | 4.02 | 0.47 | 0.862 |
| *unclassified_Prevotellaceae* | 2.48 | 3.48 | 0.43 | 0.157 |
| *Faecalibacterium* | 2.24 | 3.00^*^ | 0.27 | 0.045 |
| *[Ruminococcus]_torques_group* | 2.66 | 1.99 | 0.43 | 0.306 |
| *uncultured_rumen_bacterium* | 2.68 | 1.94 | 0.36 | 0.169 |
| *Phascolarctobacterium* | 1.75 | 2.53^*^ | 0.24 | 0.042 |
| *NK4A214_group* | 1.78 | 2.41 | 0.36 | 0.274 |

Note: Values with superscripts "*" indicate significant difference (*P*<0.05), "**" indicate significant difference (*P*<0.01).
