# Supplementary material for: Effects of supplementation with vitamin D3 on growth performance, lipid metabolism and cecal microbiota in broiler chickens
Source: Front Vet Sci. 2025 Feb 6;12:1542637. doi: 10.3389/fvets.2025.1542637 (PMC11839666; doi:10.3389/fvets.2025.1542637)
Supplement: Supplementary file 3 [file Table_3.docx]

***Supplementary Material***

**Table S3.** Effect of dietary VD_3_ microbiota composition of the cecum at genus level of broilers at 84 days of age.

| **Item** | **CON group (%)** | **VD group (%)** | **SEM** | **P-Value** |
| --- | --- | --- | --- | --- |
| *Bacteroides* | 19.24 | 23.28^*^ | 1.01 | 0.018 |
| *Rikenellaceae_RC9_gut_group* | 8.99 | 10.01^*^ | 0.31 | 0.039 |
| *uncultured_rumen_bacterium* | 4.83 | 3.01^*^ | 0.42 | 0.011 |
| *uncultured_Verrucomicrobia_bacterium* | 5.45 | 1.69^**^ | 0.37 | <0.001 |
| *Parabacteroides* | 3.30 | 2.97 | 0.35 | 0.519 |
| *Phascolarctobacterium* | 2.97 | 2.61 | 0.40 | 0.534 |
| *unclassified_Bacteroidales* | 1.88 | 3.59^*^ | 0.41 | 0.015 |
| *Lachnoclostridium* | 1.62 | 3.32^**^ | 0.28 | 0.002 |
| *Desulfovibrio* | 2.29 | 2.29 | 0.23 | 0.983 |
| *Faecalibacterium* | 1.53 | 2.90^**^ | 0.14 | <0.001 |

Note: Values with superscripts "*" indicate significant difference (*P*<0.05), "**" indicate significant difference (*P*<0.01).
