# Supplementary material for: Effects of supplementation with vitamin D3 on growth performance, lipid metabolism and cecal microbiota in broiler chickens
Source: Front Vet Sci. 2025 Feb 6;12:1542637. doi: 10.3389/fvets.2025.1542637 (PMC11839666; doi:10.3389/fvets.2025.1542637)
Supplement: Supplementary file 5 [file Image_1.pdf]

Supplementary Material

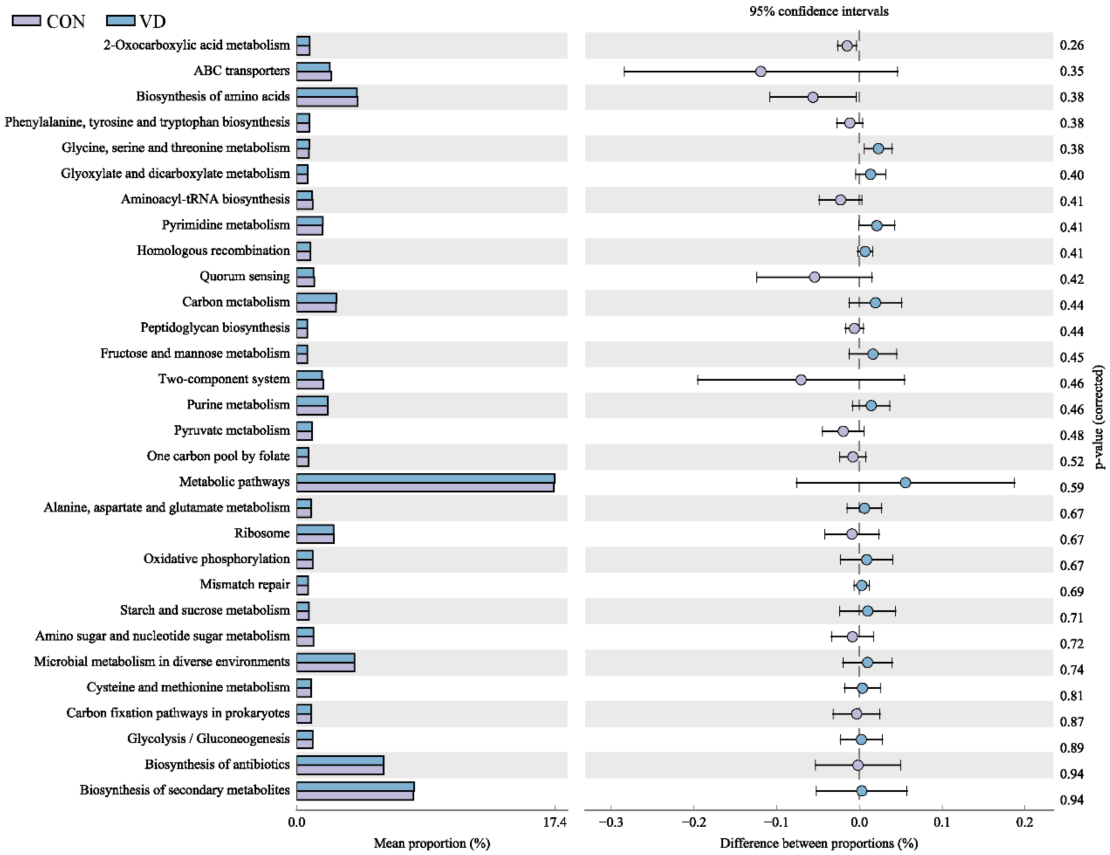

Supplementary Figure S1. Differential KEGG level 3 metabolic pathways between groups (at 56 days). The p-value on the right is derived from G-test in the statistical analysis of taxonomic and functional profiles (STAMP) software, where p-value <0.05 indicates a significant difference.
